# Supplementary material for: A neural network for information seeking
Source: Nat Commun. 2019 Nov 14;10:5168. doi: 10.1038/s41467-019-13135-z (PMC6856099; doi:10.1038/s41467-019-13135-z)
Supplement: Supplementary file 1 — Supplementary Information [file 41467_2019_13135_MOESM1_ESM.pdf]

Supplementary Information

A neural network for information seeking

White et al. (J. Kael White, Ethan S. Bromberg-Martin, Sarah R. Heilbronner, Kaining Zhang, Julia Pai, Suzanne N. Haber, Ilya E. Monosov)

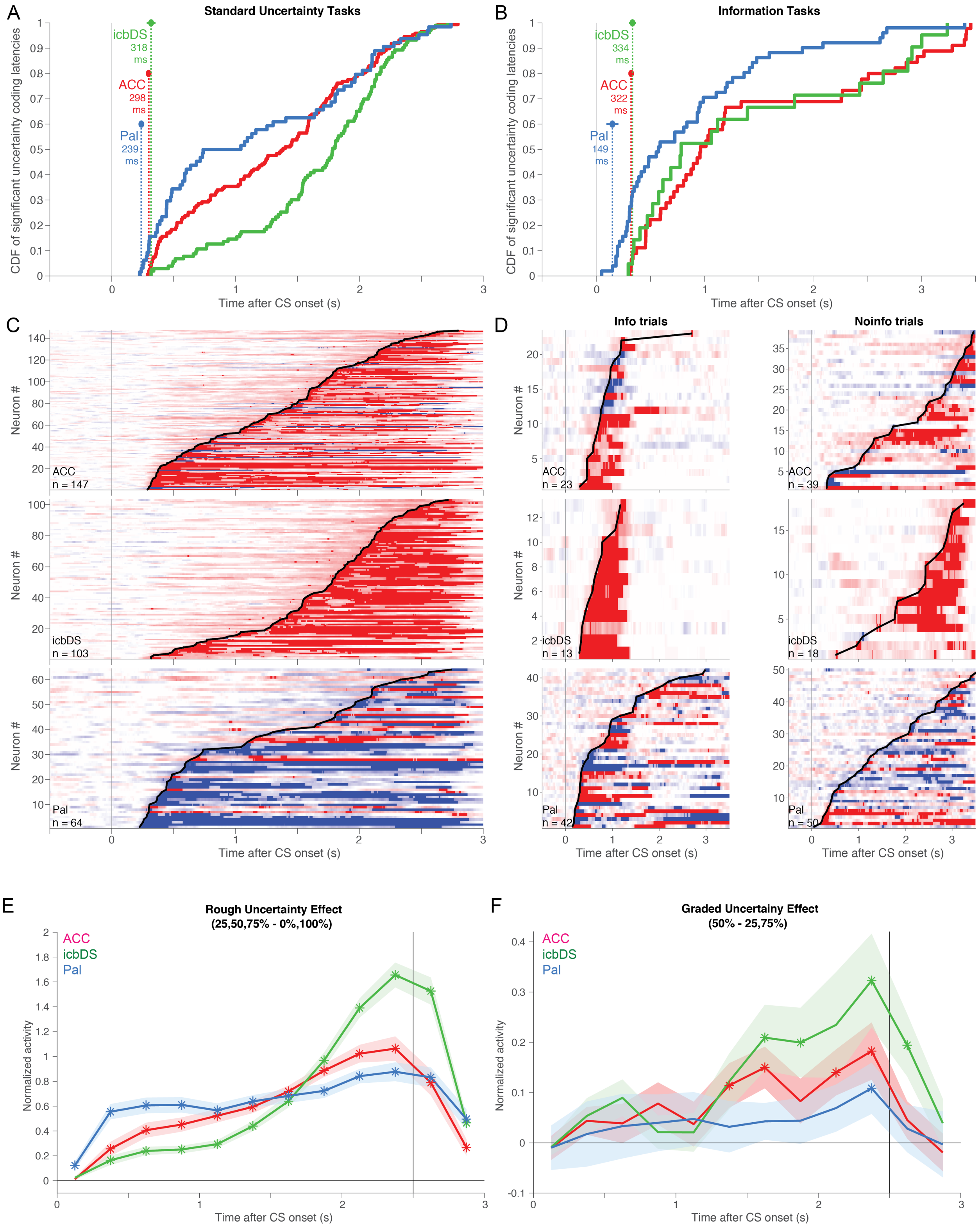

Supplementary Figure 1. Detailed properties of uncertainty signals: latency, sign, and emergence of rough vs. graded coding.

(A) Cumulative distribution of latencies for all single neurons recorded during standard uncertainty tasks that had detected latencies of uncertainty coding. Circles, dashed vertical lines, and text indicate the estimated population latency of uncertainty coding in each area (error bars are ± 1 bootstrap SE). Uncertainty signals emerge first in Pal, followed by ACC and icbDS at similar latencies. The population latency is significantly shorter in Pal than both ACC and icbDS ($p = 0.005$ and $p = 0.001$, permutation tests) and not significantly different between ACC and icbDS ($p = 0.35$).
(B) Same, for neurons recorded during information tasks. Latencies were calculated separately for Info and Noinfo trials, and each neuron's overall latency of uncertainty coding was defined as the shorter of the two. Note that the power to detect latency differences between areas is considerably smaller in this dataset due to the smaller number of neurons. Nonetheless, the data shows a similar pattern: the population latency is significantly or trending to be shorter in Pal than both ACC and icbDS ($p = 0.018$ and $p = 0.15$) and not significantly different between ACC and icbDS ($p = 0.63$).
(C) Heat map of uncertainty coding over time for the neurons in (A). Top: ACC; middle: icbDS; bottom: Pal. Each row is a neuron. Black lines indicate each neuron's detected latency of uncertainty coding. Color indicates the neuron's uncertainty coding at each time during the task (red = more active for uncertain than certain CSs, blue = less active for uncertain than certain CSs, white = no uncertainty coding; dark colors = significant uncertainty coding, light colors = non-significant uncertainty coding (darkest possible light blue means ROC = 0, darkest possible light red means ROC = 1)). Uncertainty coding is quantified based on the ROC area for using the neuron's activity to discriminate between uncertain vs certain CSs. For this latency analysis we conservatively calculate the uncertainty coding at each time point as the least extreme of two ROC areas: one for discriminating all uncertain CSs from the 100% reward CS, and the other for discriminating all uncertain CSs from the 0% reward CS. Uncertainty coding is predominantly excitatory in ACC, almost exclusively excitatory in icbDS, and predominantly inhibitory in Pal.
(D) Same, for neurons recorded during information tasks. Data is shown separately for Info and Noinfo trials (left and right columns). For this analysis we calculate the uncertainty coding at each point as the ROC area for discriminating uncertain from certain reward trials. Note that the number of neurons is different because neurons with detectable latencies on Info trials did not always have detectable latencies on Noinfo trials, and vice versa.
(E) Population average rough uncertainty signal for each area during standard uncertainty tasks, defined as the difference in mean normalized activity between uncertain CSs (25%,50%, and 75%) and certain CSs (0% and 100%). The shaded area is ± 1 SE. Asterisks indicate significant differences from 0 (signed-rank test, $p < 0.05$). Consistent with the latency analysis, the rough uncertainty signal reaches significance first in Pal and later in ACC and icbDS. The population average rough uncertainty signal was significantly higher in Pal than either ACC or icbDS in all time bins within the first 1 sec after CS onset (all $p < 0.05$, rank-sum tests), and conversely, was significantly higher in both ACC and icbDS than Pal in all time bins in the last 0.75 sec before outcome delivery (all $p < 0.05$, rank-sum tests). This indicates that the shorter latency of uncertainty signals in Pal was due to Pal having a different timecourse of uncertainty coding, consisting of faster but ultimately weaker signals (i.e., not due to Pal simply having overall stronger signals than other areas, or due to Pal having the same average signal strength as other areas but concentrated into small fraction of its uncertainty coding neurons that are endowed with especially strong signals that allow their latencies to be detected earlier).

(F) Population average graded uncertainty signal in each area, defined as the difference in mean normalized activity between the maximally uncertain CS (50%) and the intermediately uncertain CSs (25% and 75%). There is a trend for a graded uncertainty coding at all time points in all areas, but it grows and reaches significance first in ACC and icbDS, and later in Pal. Thus, while rough uncertainty coding emerged first in Pal, graded uncertainty coding became significantly prevalent first in ACC and icbDS.

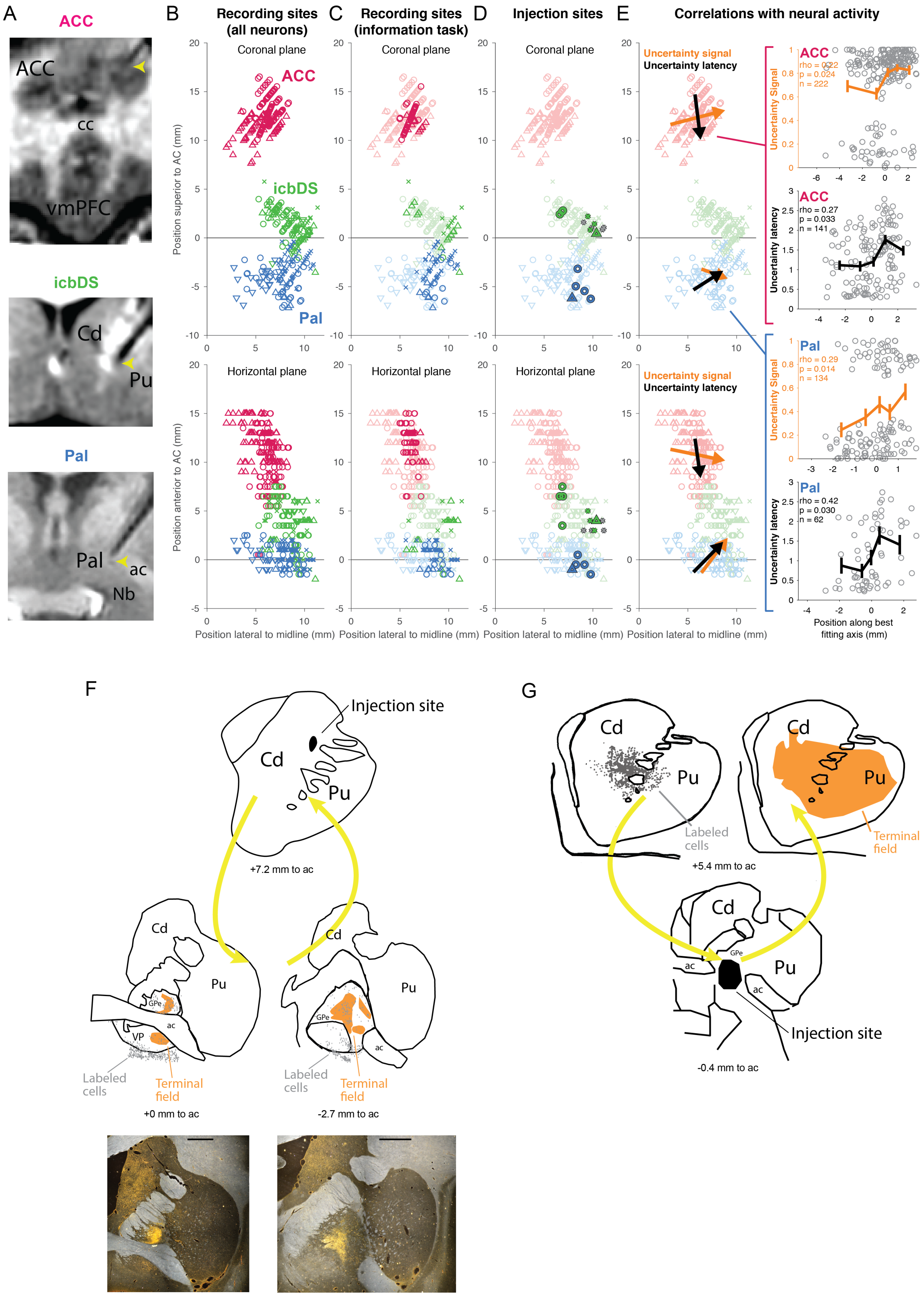

Supplementary Figure 2. Anatomical tracing, reconstruction of recording sites, and correlation with neural responses.

(A) MRI verification of recording sites. MRIs were taken immediately after recording uncertainty coding neurons with the electrode still in place. Shown are coronal views in which the electrode track is visible as a black ‘shadow’ on the MRI; the yellow arrow indicates the

location of the electrode tip. Top: Recording site in ACC, at a location symmetrical to the ACC in the opposite hemisphere. Middle: Recording site in icbDS, at a location intermediate between the body of the caudate and putamen. This scan has been reported previously[1]. Bottom: Recording site in Pal, with the tip adjacent to the ventral boundary of the anterior commissure. Abbreviations: cc, corpus callosum; vmPFC, ventromedial prefrontal cortex; Cd, caudate; Pu, putamen; ac, anterior commissure; Nb, nucleus basalis.
(B) Anatomical locations of recording sites. Reconstructed 3D coordinates of each neuron in the dataset, shown for all areas (indicated by colors) and all animals (indicated by symbols: animals B, R, W, and Z are circles, crosses, downward triangles, and upward triangles). Coordinates are relative to the midline, superior tip of the anterior commissure (AC). *Top* shows coordinates in the coronal plane. *Bottom* shows coordinates in the horizontal plane. The three areas where uncertainty-responsive neurons were found were clearly anatomically distinct from each other, and were located in similar, overlapping locations in all animals.
(C) Anatomical locations of recording sites for each task type. Light colors indicate neurons recorded in standard uncertainty tasks. Dark colors indicate neurons recorded in information tasks. There is considerable overlap between recording sites for the two task types.
(D) Anatomical locations of injection sites. Same as (B) with an overlay showing the reconstructed coordinates of the injection sites. Colors indicate injection type (green, icbDS muscimol; blue, Pal muscimol; gray, icbDS saline). The injection sites for icbDS and Pal were clearly distinct from each other and overlapped the locations of uncertainty-responsive neurons in their respective areas. The injection sites for saline overlapped with the injection sites for muscimol in the same area.
(E) Anatomical relationships to neural responses. We plot all significant correlations between neuron locations and response properties ($p < 0.05$, permutation test). Each relationship is indicated by an arrow in the left column (viewed in the coronal plane (top) and in the horizontal plane (bottom)), and a corresponding scatterplot on the right column.
Left: Colored arrows in each area indicate relationships between neuron location and one of four response properties: uncertainty signal (orange), uncertainty signal latency (black), fitted gaze-modulation latency (none significant), fitted gaze-modulation gain change expressed in $\log_2$ units (none significant). Each arrow passes through the center of mass of the locations of the neurons recorded in that area, and is directed along the vector in 3D space that best predicts the response property. Specifically, we fitted each response property with linear regression as a function of neural coordinates in 3D space (ResponseProperty = $\beta_0 + \beta_1$*Lateral + β_2*Anterior + β_3*Superior + ε). We then pointed the arrow in the direction of the unit vector $v = \langle\beta_1, \beta_2, \beta_3\rangle / |\langle\beta_1, \beta_2, \beta_3\rangle|$, and scaled the arrow's length by the standard deviation of the Euclidean distance between the neuron locations and their center of mass. P-values and significance were assessed with a permutation test (by comparing the log likelihood of the fit to the true dataset with the distribution of log likelihoods of fits to 20000 permuted datasets generated by shuffling the relationship between the 3D coordinates of neurons and the responses of those neurons).
Right: Each plot shows a single significant relationship. Each dot is a single neuron's response property. The x-axis indicates each neuron's location relative to its area's center of mass, projected onto the best-predictive unit vector for that area. The y-axis indicates that neuron's response property in the task. Lines with five data points indicate mean ± SE of the response property for the neurons that were located in each quintile of the neuron locations along the best-fit line. Text indicates rank correlation, the p-value of the linear regression that determined the vector (permutation test described above), and the number of neurons for the comparison.

Uncertainty latency relationships have fewer neurons than other response properties because not all neurons had detectable latencies (Supplementary Fig 1).

In ACC, there were tendencies for superior-lateral neurons to have stronger uncertainty signals (orange, $p = 0.024$) and for posterior-inferior neurons to have longer latencies of uncertainty signals (black, $p = 0.033$). In Pal, there were tendencies for anterior-lateral neurons to have stronger uncertainty signals (orange, $p = 0.014$) and longer latencies of uncertainty signals (black, $p = 0.030$). Certain neuronal signals and functions of the ACC may be organized in a dorsal-ventral manner (e.g. based on their location across the dorsal and ventral banks of the cingulate sulcus). Therefore, we explicitly tested whether the coding properties of ACC neurons varied significantly with their position along the dorsal-ventral axis. Consistent with our analysis in Supplementary Fig 2E, there was a significant correlation such that uncertainty coding neurons located more dorsally had more positive uncertainty signals ($n = 222$, rho = +0.16, $p = 0.017$). There were no significant correlations with other coding properties (uncertainty coding latency in neurons with detectable latencies, $n = 141$, rho = 0.02, $p = 0.795$; gaze-modulation latency, $n = 221$, rho = -0.02, $p = 0.729$; gaze-modulation gain factor, $n = 221$, rho = 0.00, $p = 0.978$).

(F) Additional tracer injection into icbDS confirms bidirectional icbDS-Pal projections. Top: the tracer fluororuby was injected into the internal capsule-bordering region of the dorsal striatum (black area, top). Retrogradely labeled cell bodies (gray dots, bottom) and anterogradely labeled terminal fields (orange shaded areas, bottom) were found in Pal, including both the ventral pallidum (below the anterior commissure) and the anterior globus pallidus external segment (above the anterior commissure). Text indicates the anterior-posterior position relative to the midline anterior commissure. Bottom: photomicrographs show anterogradely labeled terminal fields in Pal. Scale bar: 2 mm.
(G) Tracer injection into Pal confirms bidirectional icbDS-Pal projections. The tracer Lucifer Yellow was injected into the border of the globus pallidus external segment and ventral pallidum (black area, bottom). Retrogradely labeled cell bodies (gray dots, top left) and anterogradely labeled terminal fields (orange shaded areas, right) were found in the dorsal striatum, including the internal capsule-bordering regions of the caudate and putamen. Text indicates the anterior-posterior position relative to the midline anterior commissure. Abbreviations: Cd, caudate nucleus; Pu, putamen; ac, anterior commissure; GPe, globus pallidus external segment; VP, ventral pallidum.

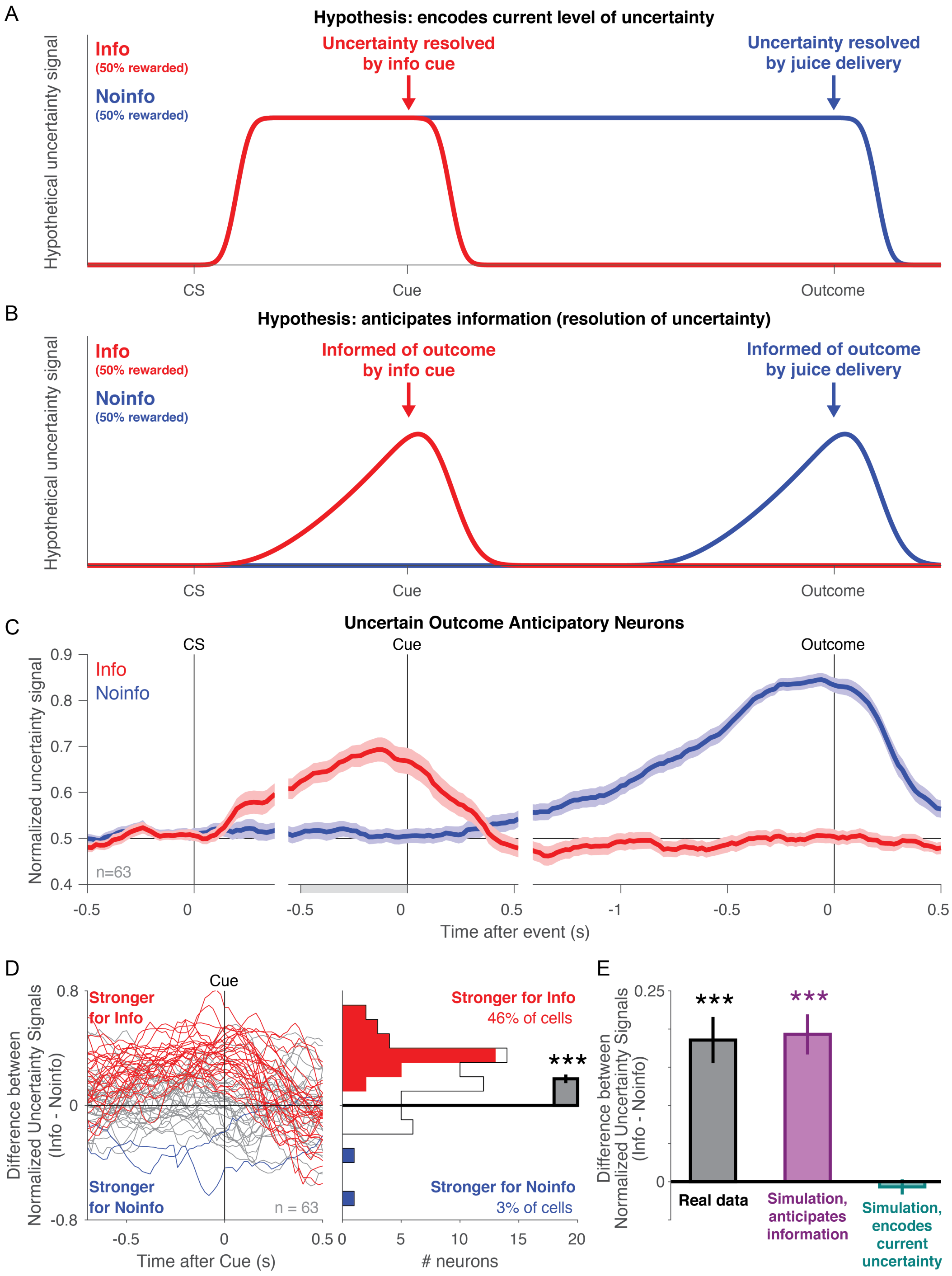

Supplementary Figure 3. Information-anticipatory activity cannot be explained by encoding of the current level of uncertainty.
(A) Hypothetical timecourse of uncertainty signals for a neuron encoding the animal's current level of uncertainty about the size of the trial's reward outcome. The neuron would respond equally to both the Info 50% reward CS and Noinfo 50% reward CS, because the reward is equally uncertain in both situations (i.e. they both predict identical reward distributions: 50% chance of reward, 50% chance of no reward). The only difference between the conditions would

occur after the CS period ended, based on the time the neuron terminated its response. On Info trials, the neuron would stop responding after the Info cue (because the cue informs the animal of the upcoming reward, and hence fully resolves the uncertainty), but on Noinfo trials, the neuron would only stop responding after outcome delivery (because the cue does not predict the outcome, and hence the uncertainty is only resolved after outcome delivery).
(B) Hypothetical timecourse of uncertainty signals for a neuron that anticipates the time of receiving information to resolve reward uncertainty. Same as Fig 2B. The neuron should clearly differentiate between Info and Noinfo 50% reward CSs during the CS period, with a stronger uncertainty signal for the Info 50% reward CS.
(C) Observed timecourse of normalized uncertainty signals in the uncertainty coding neurons that had significant Uncertain Outcome Anticipation Indexes. Same as Fig 3C. Importantly, these neurons were not selected in any manner based on their activity on Info trials or their activity during the CS period; they were only selected based on their outcome-anticipatory activity on Noinfo trials. Nonetheless, these neurons clearly differentiated between Info and Noinfo trials during the CS period, with stronger uncertainty signals during the Info CS.
(D) Difference between timecourses of normalized uncertainty signals on Info trials vs. Noinfo trials (i.e., red curve in panel C – blue curve in panel C) for each of these neurons. Red, blue, and gray lines indicate neurons with significantly positive, negative, or non-significant differences ($p < 0.05$, permutation test on the activity in a 500 ms window before cue onset). A large fraction of the neurons have higher normalized uncertainty signals for Info, in a manner that ramps up to the time of the cue.
(E) Quantification of the mean difference between normalized uncertainty signals in the 500 ms before cue onset. Red, blue, and white cells correspond to the colors in panel D. Nearly half of cells have significant positive differences (46% of cells, higher than expected by chance, $p < 0.001$, binomial test). Only 2 neurons have significant negative differences (3% of cells, not significantly different from chance, $p = 0.47$). The average difference over all neurons is +0.186, which is significantly greater than 0 (gray bar, $p < 0.001$, signed-rank test).
(F) Direct test of whether neural activity is better explained by anticipation of information or encoding of the current level of uncertainty. Bars indicate the mean ± SE of the mean differences plotted in panels D,E. The gray bar represents this value computed using the real data (same as the gray bar in panel E). The other bars represent this value computed using simulated datasets representing the two competing hypotheses. The simulated datasets for each hypothesis were generated by starting with the real dataset, finding pairs of task conditions that *should* have equivalent activity according to that hypothesis, and by drawing new firing rates for each trial in the first condition from the distribution of firing rates in the second condition, to force those conditions to be equivalent in the simulated data. This procedure was done separately for each neuron. For the simulations representing 'anticipates information', activity during the CS period on Noinfo trials should be equal to activity on Info certain trials (i.e. little or no activity). Hence we set the rates on all Noinfo trials to be drawn from the distribution of rates on Info certain trials. For the simulation representing 'encodes current uncertainty', Info and Noinfo trials should have equivalent activity during the CS period. Hence we set the rates on Info certain trials to be drawn from the distribution of rates on Noinfo certain trials, and set the rates of Info uncertain trials to be drawn from the distribution of rates on Noinfo uncertain trials. The results were clear: the mean difference in the real data was highly similar to the mean difference in the simulated 'anticipates information' dataset (purple bar), and clearly distinct from the simulated 'encodes current uncertainty' dataset (blue bar).

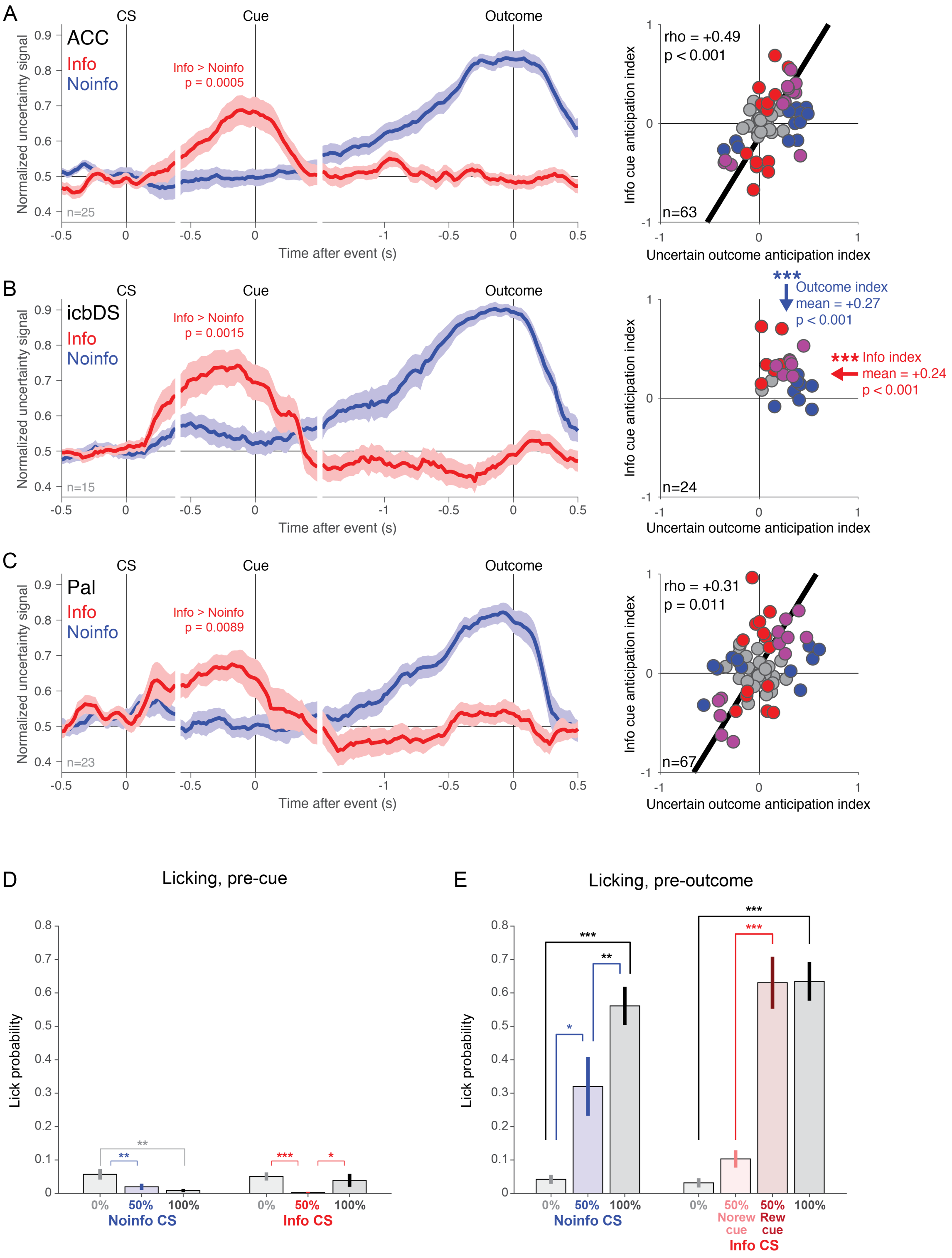

Supplementary Figure 4. Information-anticipatory activity in all areas, and absence of information-anticipatory licking behavior.

(A,B,C) Test for information-anticipatory activity in ACC (A), icbDS (B), and Pal (C). *Left:* same format as Fig 3C for each area. The average uncertainty signal of neurons that had a significant ramping uncertainty activity measured only using Noinfo trials (i.e. cells with a

significant Uncertain Outcome Anticipation Index, $p < 0.05$, permutation test). Note that these neurons are selected solely based on their activity anticipating the outcome on Noinfo trials. Even so, in all areas these populations show a strong uncertainty signal in the same direction anticipating the cue on Info trials. Thus, these neural populations had information-anticipatory activity resembling the theoretical pattern in Fig 2B.
Right: same format as Fig 3D for each area. Best-fit lines from type 2 regression are plotted for all areas with significant correlations ($p < 0.05$); arrows indicate significant mean indexes different from 0 ($p < 0.05$). All areas show coding indexes consistent with information-anticipatory activity, though in different manners due to the different signs of neural coding in icbDS vs ACC and Pal. icbDS shows an especially strong pattern of information-anticipatory activity. icbDS neurons only encode uncertainty with a positive sign, and nearly all neurons are in the upper right quadrant, indicating positive anticipation of both informative cues and uncertain outcomes (mean Informative Cue Anticipation Index = +0.24, mean Uncertain Outcome Anticipation Index = +0.27; both significantly greater than 0, signed-rank tests, both $p < 0.0001$). These two indexes are generally consistent across neurons with relatively low variability so there is no significant correlation between them (rho = -0.27, $p = 0.195$). By contrast, ACC and Pal populations included subsets of neurons with different signs and variable strengths of uncertainty coding in standard uncertainty tasks (Supplementary Fig 1). As a result, information-anticipatory activity should not necessarily lead to non-zero population average indexes, but should result in the two indexes being correlated, indicating that individual neurons anticipate both informative cues and uncertain outcomes in similar manners. Indeed, in both areas there is a strong and significant correlation such that cells with a more positive Informative Cue Anticipation Index also have a more positive Uncertain Outcome Anticipation Index (ACC: $p < 0.001$; Pal: $p = 0.011$) – the same pattern seen in Fig 3D for the population of all uncertainty-responsive neurons across the network.
(D) Mean fraction of trials of each type in which a lick was detected in the 500 ms before cue onset. Data are from n=18 sessions in which licking was measured and there was reliable differential licking that distinguished 100% reward from 0% reward trials. Error bars are ± 1 SE. Licks occurred before the cue on ~5% or fewer trials, indicating that animals had little or no expectation that juice would be delivered at the time of the cue. Notably, the Info 50% reward CS evoked intense gaze and neural activity in anticipation of the informative cue (Fig 2-4), but evoked near-zero licking, indicating that the information-related behavior and neural activity cannot be accounted for by expectation of juice reward. If anything, there was slightly but significantly less licking for the Info 50% reward CS than for the other Info CSs. There were significant but small differences in licking between some other conditions as well (Noinfo 50% vs 0%, $p = 0.005$; Info 50% vs 0%, $p = 0.001$; Info 50% vs 100%, $p = 0.032$).
(E) Same as (A), for the 500 ms before outcome delivery. Licks occurred on a large fraction of trials, indicating that animals expected juice to be delivered at the time of the outcome. Licking was generally consistent with the mean reward in each condition. On Noinfo trials licking significantly increased with reward probability (100% > 50%, $p = 0.003$; 50% > 0%, $p = 0.044$; signed-rank tests). On Info trials licking was significantly greater after reward was cued than after no-reward was cued, both for trials in which reward was initially uncertain (50%→reward > 50%→no reward, $p = 0.001$) and trials when reward was initially certain (100% > 0%, $p < 0.001$).

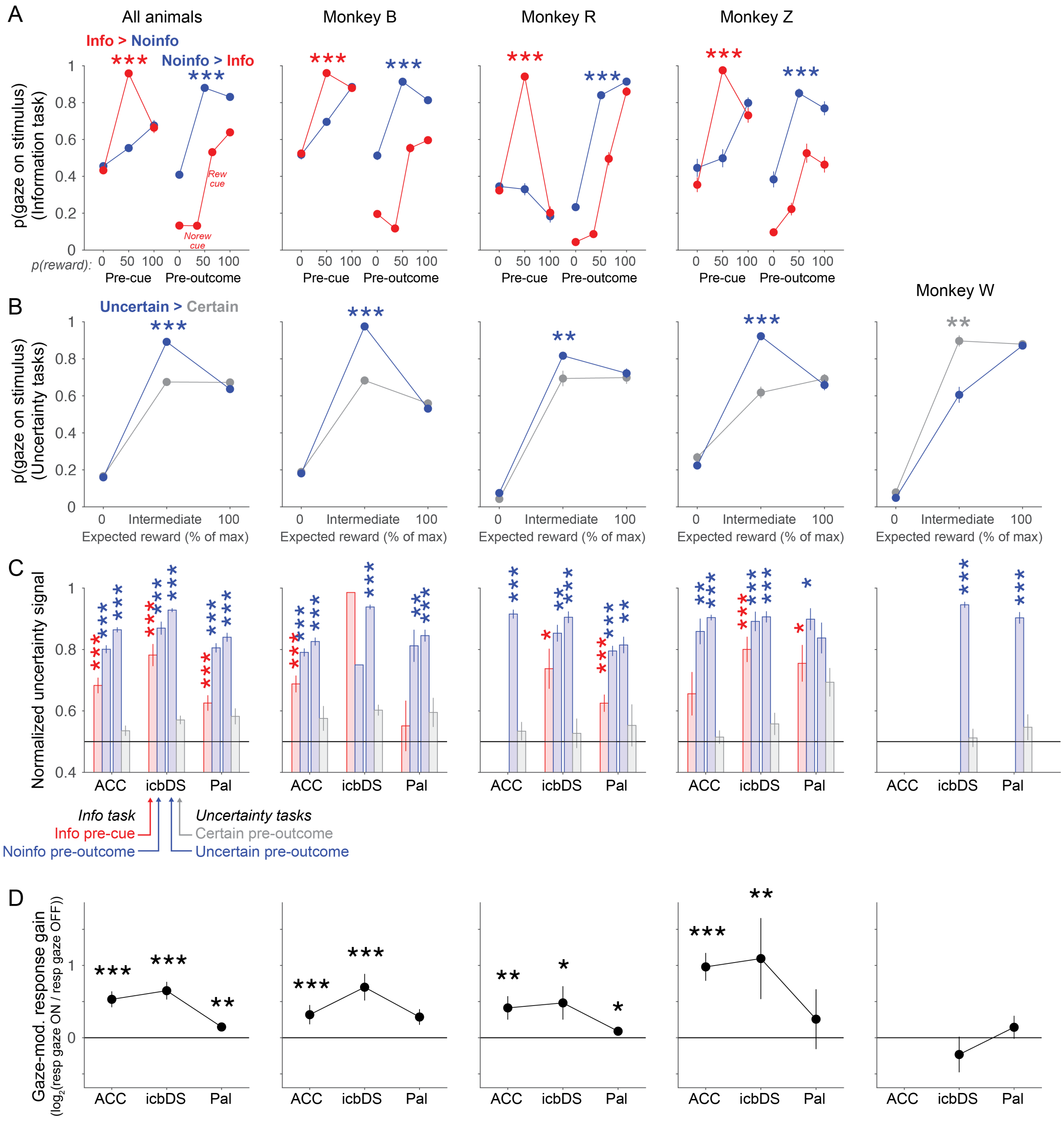

Supplementary Figure 5. Gaze behavior and neural activity for each animal and area. The three animals that contributed the great majority of the data – animals B, R, and Z – showed strong and consistent information-anticipatory behavior in the information task (A) and standard uncertainty tasks (B); showed strong and consistent information-anticipatory neural signals in ACC, icbDS, and Pal (C); and showed similar and consistent links between fluctuations in their gaze behavior and fluctuations in the gain of their information-related neural signals (D). In addition, we were able to find a single animal (animal W) that lacked normal uncertainty-related gaze behavior, and were able to collect neural data from BG in the standard uncertainty task (B). This animal appeared to have neural uncertainty signals with the usual magnitude (C) but they lacked the usual coupling to gaze behavior (D).

(A) Gaze behavior in the information task, averaged over all animals (left column) and in each individual animal (other columns). The fraction of the time spent with gaze on the stimulus is plotted for each Info (red) and Noinfo (blue) CS and cue condition in 500 ms time windows before the cue (left) and outcome (right). Info 50% CS pre-outcome gaze is plotted separately for trials when the no-reward cue (left data point) or reward cue (right data point) was shown. Error bars are ± SE. The pooled data from all animals shows clear information-anticipatory gaze behavior, with greater gaze on trials with uncertain outcomes than certain outcomes, significantly greater gaze pre-cue in anticipation of informative than non-informative cues (left, 50% Info CS (red) > 50% Noinfo CS (blue); *** indicates $p < 0.001$, signed-rank test) and significantly greater gaze pre-outcome in anticipation of uninformed than already-informed outcomes (right, Noinfo cues following Noinfo 50% CS (blue) > Info cues following Info 50% CS (red); $p < 0.001$). Similar behavior occurred in all three animals (B, R, and Z).

(B) Gaze behavior in standard uncertainty tasks as a function of the expected reward value of the CSs, for blocks of trials where intermediate-value CSs were associated with uncertain rewards (blue, e.g. 50% chance of 0.25 mL or 0 mL juice) or certain rewards with equal expected value (gray, e.g. 100% chance of 0.125 mL juice). Gaze was measured in a 500 ms time window before the outcome. Animals B, R, and Z had significantly higher gaze at uncertain CSs than at certain CSs with the same expected value (*, **, *** indicate $p < 0.05, 0.01, 0.001$, signed-rank tests), consistent with their information-anticipatory behavior in the information task. By contrast, animal W had significantly lower gaze at uncertain than certain CSs.

(C) Population average neural uncertainty signals in all animals, areas, and tasks. Uncertainty signals in each area are quantified as ROC areas for distinguishing between task conditions, as follows. Information task: Bar 1 (red), pre-cue Info uncertain vs certain CSs; Bar 2 (blue), pre-outcome Noinfo uncertain vs certain CSs. Standard uncertainty tasks: Bar 3 (blue), uncertain reward blocks, pre-outcome uncertain CSs vs 0,100% CSs; Bar 4 (gray), certain reward blocks, pre-outcome intermediate value CSs vs 0,100% CSs. Asterisks indicate significance (*, **, *** indicate $p < 0.05, 0.01, 0.001$, signed-rank tests). The pooled data from all animals shows that strong and significant uncertainty signals are present in all areas both pre-cue on Info CSs trials (red) and pre-outcome on Noinfo CS trials and in uncertain reward blocks of standard uncertainty tasks (blue), and that no significant uncertainty signals are present in certain reward blocks in standard uncertainty tasks (gray). Similar patterns are found in all animals, areas, and tasks, although not all reach significance due to small samples sizes in some cases. Notably, strong, significant pre-outcome uncertainty coding was present in all animals, including those whose gaze was attracted either uncertain CSs (B, R, Z) and certain CSs (W).

(D) Median fitted gaze-modulation gain parameters in all animals and areas. Gains are expressed in log units ($\log_2$(rate when gaze is ON / rate when gaze is OFF)) and are quantified as population medians ± bootstrap SE. Asterisks indicate significance (signed-rank tests). The pooled data from all animals shows large increases in neural response gain around gaze shifts onto the CS in both ACC and icbDS, and a more modest but still highly significant increase in Pal. Similar patterns were seen in all animals whose gaze was attracted to uncertain CSs (B, R, and Z). However, animal W whose gaze was significantly less attracted to uncertain CSs lacked significant neural gain changes around the time of gaze shifts. Notably, the gain change in icbDS

was lower in animal W than in all three other animals ($p = 0.001$, 0.056, and 0.001 vs animals B, R, and Z, respectively).

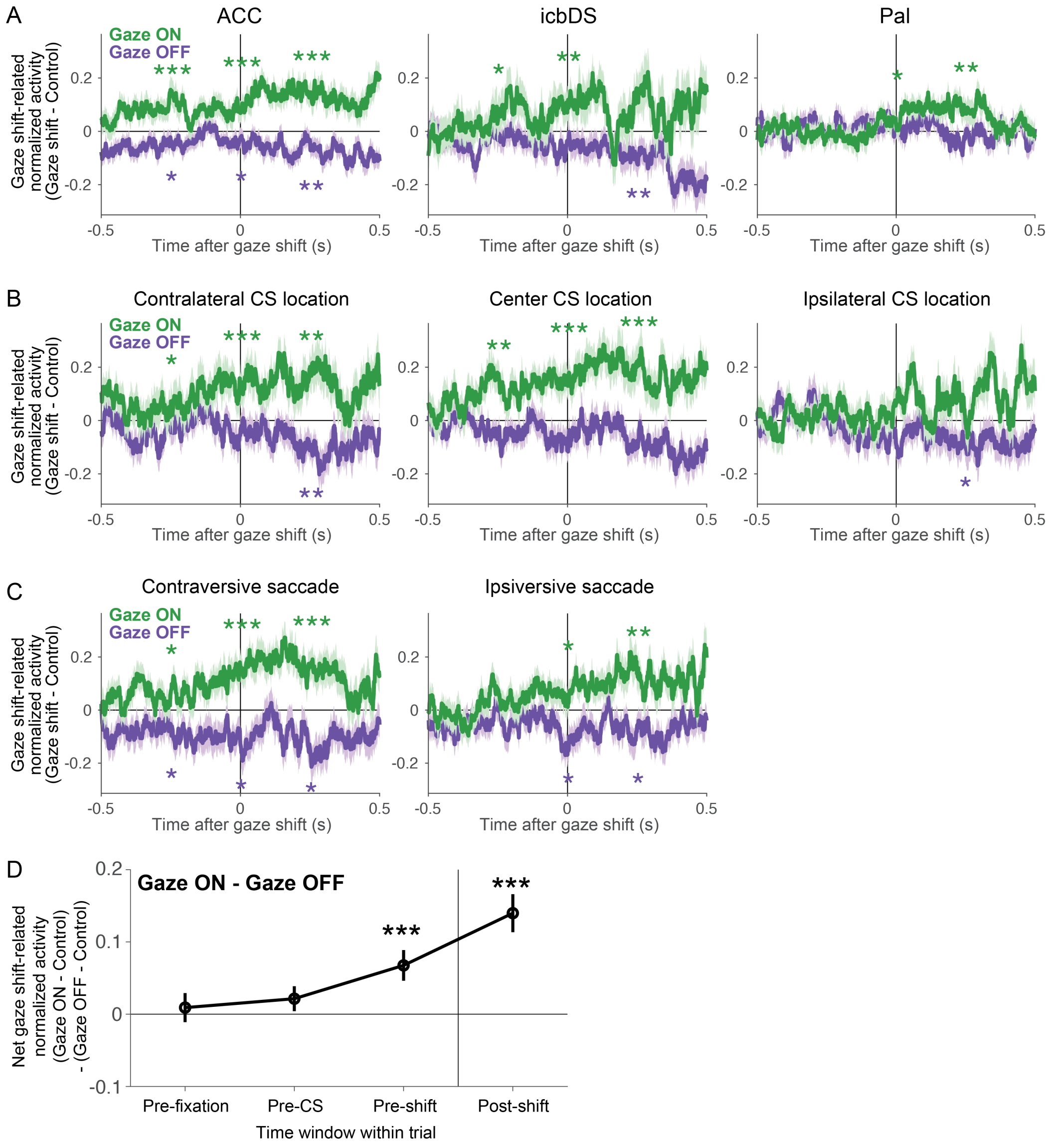

Supplementary Figure 6. Neural activity during gaze shifts for each area and gaze shift type.
(A) Population average changes in normalized activity related to gaze shifts, separately for each area. Same format as Fig 5D, but only showing data from gaze shifts when reward was uncertain (since gaze-related activity was much weaker when reward was certain (Fig 5)). Error bars are ± 1 SE. Data is shown for all neurons with at least one gaze shift onto and one gaze shift off of the stimulus, and appropriate control non-shift events for each of these shifts, when reward was uncertain (ACC n=191, icbDS n=113, Pal n=121). All areas have normalized activity that increases around gaze shifts onto the stimulus (green) and decreases around gaze shifts off of the stimulus (purple), with these effects growing over the course of the gaze shift. The effects tend to reach significance first in ACC and later in icbDS and Pal, consistent with the latencies from the model-based analysis (Fig 6). All areas at least one effect reaching significance before or during the gaze shift (i.e. too soon for effects to have been responses to altered visual feedback after the gaze shift).

(B) Population average changes in normalized activity related to gaze shifts, shown separately for CSs presented on each of the three possible positions on the screen (contralateral to the recorded neuron, center of screen, or ipsilateral to the recorded neuron). To allow valid comparisons between the curves, this plot is calculated using the n=131 neurons that could contribute data to all six curves (i.e. neurons that had one gaze shift onto the CS and off of the CS, and appropriate non-shift controls, in each of the three locations). Gaze-related activity was similar for all CS locations. There is a trend for stronger and earlier activity for gaze shifts onto contralateral and central CSs compared to ipsilateral CSs, but it did not reach significance. Importantly, if neural activity was simply encoding horizontal gaze location or stimulus location rather than motivational features of the stimuli, then activity would be substantially different after the gaze shift depending on whether the gaze was held on a CS located at the contralateral, central, or ipsilateral locations. Instead, neural activity had similar trends to be enhanced when gaze was at all the three locations, and to be suppressed after gaze shifts away from all three locations (no significant difference between locations in paired comparisons between gaze-shift related activity after the gaze shift, all $p > 0.05$ for both gaze shifts on and off the stimulus, signed-rank tests). This indicates that neural activity was not simply encoding gaze location or stimulus location.

(C) Same as (B), but split based on whether the gaze shift's saccade vector was contraversive or ipsiversive to the recorded neuron. To ensure that all gaze shifts were clearly contraversive or ipsiversive, this analysis is restricted to gaze shifts with magnitude >= 5°. Again, to allow valid comparisons between the curves, this plot is calculated using the n=134 neurons that could contribute data to all four curves. Gaze-related activity was similar for all saccade vectors. Importantly, if neural activity was simply encoding saccade execution or saccade direction rather than motivational features of the stimuli, then they should have similar saccade-related activity regardless of whether the saccade was toward or away from a CS (if encoding saccade execution) and this activity should preferentially occur for specific saccade directions (if encoding saccade direction). Instead, activity was significantly enhanced during and after all saccades toward CSs but was significantly suppressed during and after all saccades away from CSs, and this occurred regardless of whether the saccade was contraversive or ipsiversive. This indicates that neural activity was not simply encoding saccade execution or direction.

(D) Demonstration that gaze shift-related activity was closely time-locked to gaze shifts, in a manner suitable for regulating ongoing gaze behavior. This is important because we found that gaze-related activity was present for > 500 ms before and after the gaze shift (A-C). This raised the possibility that neural activity might also be related to gaze shifts over much longer timescales. For instance, neurons might have slow fluctuations in neural firing rate lasting for several minutes that covary with similarly slow fluctuations in the animal's tendency to make certain kinds of gaze shifts or to be generally engaged in the task. To test this, we calculated the mean gaze shift-related change in activity using activity in four different 500 ms time windows on each trial when the gaze shifts occurred: (1) before the onset of the fixation point, (2) before CS onset, (3) immediately before the gaze shift, (4) immediately after the gaze shift. We calculated this separately for gaze shifts onto the stimulus and gaze shifts off of the stimulus, and then calculated the difference between them (gaze shift on – gaze shift off) as a summary measure of the net strength of activity related to gaze shifts. To allow valid comparison between the data points, this analysis used the n=302 neurons that could contribute data to all data points

in all curves. The net gaze shift effect was not significantly different from zero in the pre-fixation and pre-CS time windows, but was significantly increased in the pre-gaze shift window, and significantly increased further in the post-gaze shift window. Thus, changes in neural activity were closely time-locked to upcoming gaze shifts.

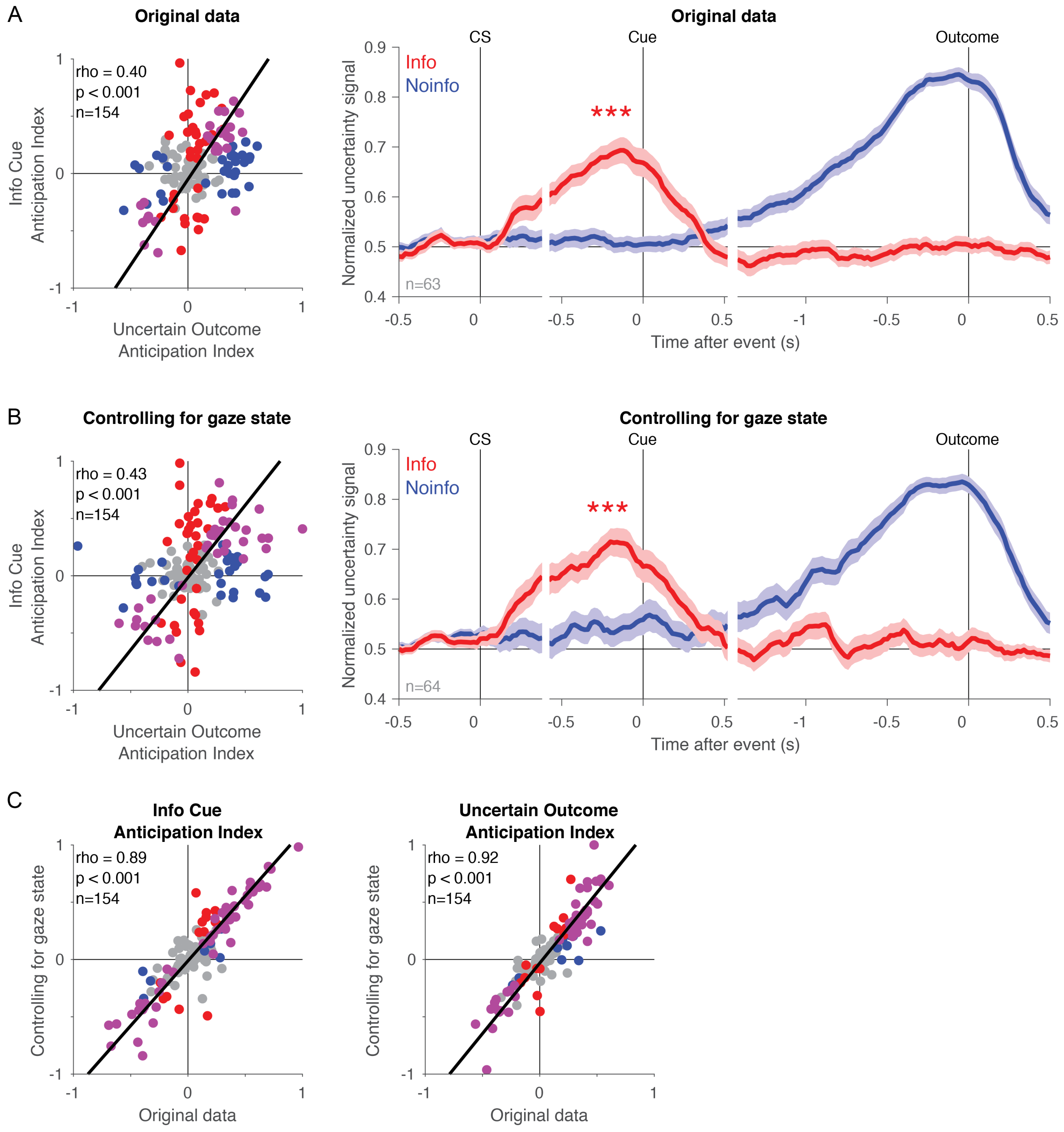

Supplementary Figure 7. Analysis of information signals while controlling for gaze state. Our analysis in Fig 5 demonstrates that while information signals are modulated by gaze, information signals are still clearly present even after neural data is split separately into times when the animal's gaze is on the stimulus vs. off the stimulus. This suggests that the presence of information signals cannot be explained as a mere side-effect of gaze-related activity. However, it remained possible that the average strength and/or timecourse of information signals (as quantified in our original analysis) might be substantially affected by the animal's gaze behavior. Animals have characteristic patterns of gazing on vs. off the stimulus at each time of the trial and on each trial type.

To control for this, we used our model of gaze-modulation from Fig 6. We used the fitted model for each neuron to remove the estimated influence of gaze on neural activity. Essentially, we adjusted each neuron's activity to be 'as if' the gaze state had been constant at all times on all

trials. Specifically, for each neuron and each millisecond of each trial, we used the neuron's fitted model to estimate $R_{real\ gaze}$, what its mean firing rate should be at each millisecond of each trial, given that trial's true time-varying gaze behavior. We also estimated $R_{constant\ gaze}$, what its mean firing rate *would have been* if the animal's gaze state had been constant on the stimulus throughout the trial. We then calculated the difference between these values, which represents the model's prediction about how gaze was affecting the neuron's firing rate at that moment in time (i.e. $R_{gaze\ effect} = R_{real\ gaze} - R_{constant\ gaze}$). Finally, we subtracted this gaze effect from the neuron's original firing rate (i.e. $R_{controlling\ for\ gaze\ state} = R_{original} - R_{gaze\ effect}$). We then repeated our analysis on this gaze state-controlled dataset.

(A) Information signals in the original dataset. Left: same as Fig 3D. Right: same as Fig 3C
(B) Information signals in the dataset controlling for gaze state. The results are highly similar to the original data. The only noticeable effect is a mild increase in uncertainty signals throughout the task, particularly at times when the animal's gaze was often away from the stimulus in the original dataset. There was little or no effect at the key moments when animals were anticipating information, as expected because the animal's gaze was already almost constantly on the stimulus at those moments in the original dataset.
(C) The Informative Cue Anticipation Index and Uncertain Outcome Anticipation Index are highly correlated between the two datasets ($\rho > 0.88$, both $p < 0.001$).

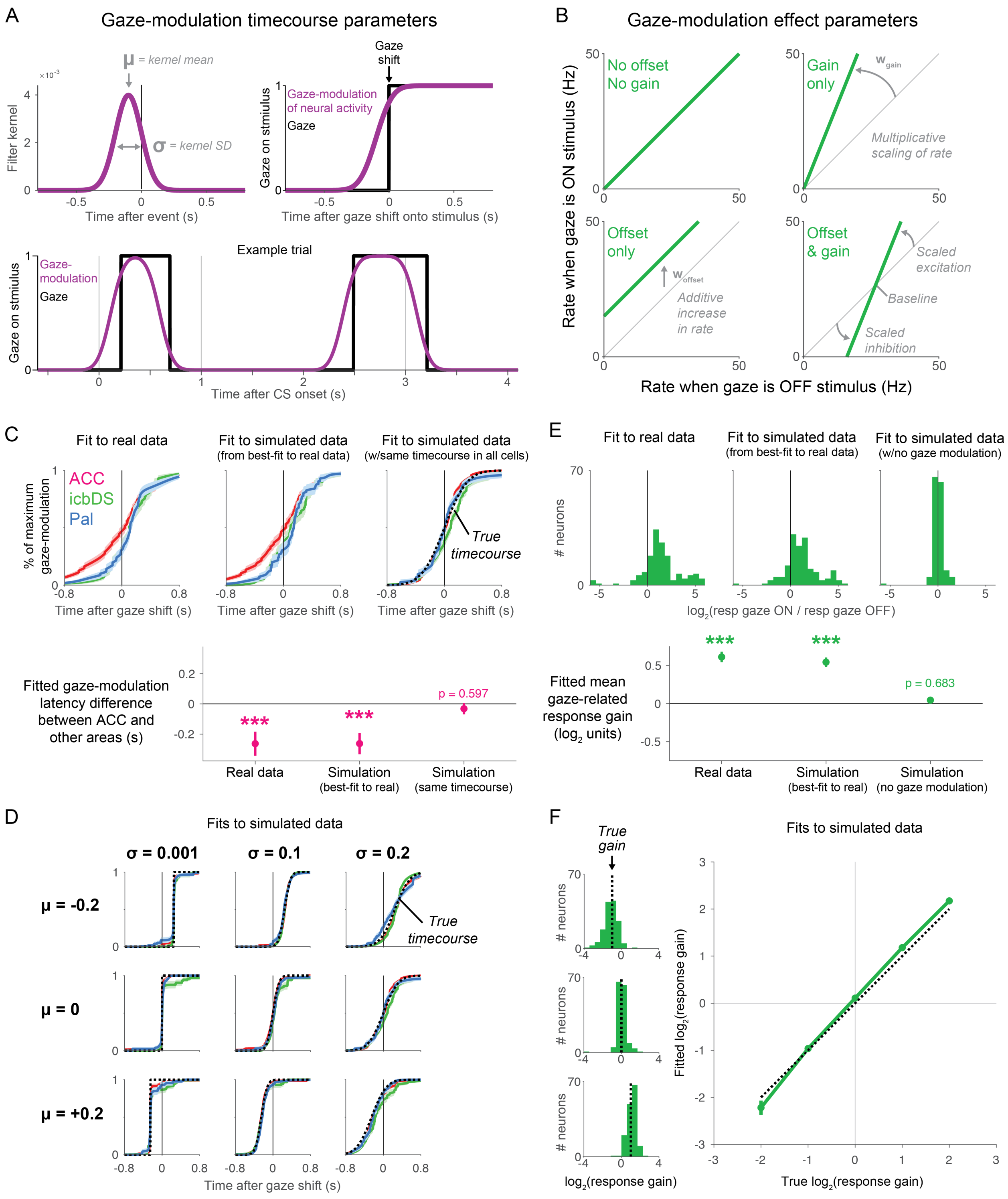

Supplementary Figure 8. Model of gaze-related modulation in single neurons. The model fitted each neuron's activity as the combination of three factors: fitted timecourse of neural response to each trial type (defined by the trial's CS and cue) when the gaze is off the stimulus, fitted timecourse of gaze-modulation from when gaze is on the stimulus (A), and fitted effect of

gaze-modulation (B). Our simulations indicate that the model fits accurately recover a close approximation of the true timecourse and effect of gaze modulation. This is true for simulations mimicking the properties of the real data (C,E), simulations representing null hypotheses that gaze effects are absent (C,E), and simulations representing alternative hypotheses that timecourses and effects of gaze modulation are different from those seen in the real data (D,F).

(A) Model parameters controlling the timecourse of gaze-modulation (μ and σ). Gaze (black) was defined at each millisecond of each trial as 1 if the eye was within 3° of the center of the trial's stimulus location and 0 otherwise. GazeMod (purple) was defined by smoothing Gaze with a Gaussian kernel specified by two parameters, the mean μ and standard deviation σ (*top left*). Thus, after a gaze shift onto the stimulus, the timecourse of GazeMod was a sigmoid, cumulative Gaussian function rising from 0 to 1, with its temporal shift controlled by μ and the gradualness of its rise controlled by σ (*top right*). For example, during the same example trial shown in Fig 5, the model's GazeMod goes up and down in temporal alignment with changes in Gaze (*bottom*). GazeMod then controls the degree to which firing rate is affected by the parameters in (B). For instance, when GazeMod = 0 firing rate is unaffected by gaze; when GazeMod = 0.5 firing rate is affected half-maximally by gaze; when GazeMod = 1 firing rate is maximally affected by gaze.

(B) Model parameters controlling the effect of gaze-modulation (w_{gain} and w_{offset}). These allow GazeMod to cause a multiplicative scaling of neural responses (w_{gain}), an additive increase or decrease in neural activity (w_{offset}), or both. Specifically, the model's firing rate is specified by equation (2), which is equivalent to: $Rate = Rate_{GazeOFF}$*(1 + GazeMod*w_{gain}) + GazeMod* w_{offset}. Thus, if gaze is on the stimulus, it has different effects on firing rate depending on these two parameters. If $w_{gain} = 0$ and $w_{offset} = 0$ then firing rate is unaffected (*top left*). If $w_{gain} > 0$, neural activity is multiplicatively scaled up in proportion to w_{gain} (*top right*). If $w_{offset} > 0$, neural activity is additively increased by w_{offset} (*bottom left*). Finally, if both w_{gain} and w_{offset} are non-zero, then neural responses (i.e. deviations in neural firing rate from a baseline level) are multiplicatively scaled up in proportion to w_{gain} (*bottom right*).

(C) Model performance: fitted timecourses of gaze-modulation for real and simulated data. Fits are shown for the real data (*left*), a simulated dataset generated from the best-fitting model to the real data (*middle*), and a second simulated dataset generated from the same model except that its gaze-modulation timecourses are forced to be the same, identical curves for all neurons in all areas (*right*, enforced timecourse indicated by black dashed curve). Simulated data was generated from a given model by calculating its predicted mean spike count for each time bin of each trial, then drawing the corresponding simulated spike count from a Poisson distribution with that mean. Fitting results are displayed as the average over all neurons that were significantly modulated by gaze in the real data. Error bars are ± 1 SE.

The fit to the real data indicates that gaze-modulation begins earlier in ACC than icbDS or Pal (*left*). The fits to the simulated data indicate that the model-fitting procedure is effective at recovering the true timecourse of gaze-modulation. Thus, in a simulation where the true timecourse is known to be equal to the curves on the left, the fits accurately recover a close approximation of those curves (compare *middle* vs *left*), including a similarly significantly earlier latency in ACC than icbDS and Pal (*bottom*). Likewise, in a simulation where the true timecourse is known to be identical in all neurons in all areas, the fits in all areas accurately

recover a close approximation of that timecourse (*right*, compare colored curves to black curve), resulting in no difference in latency between areas (*bottom*).

(D) Model performance: fitted timecourses for simulated data with a variety of true timecourses. As in the right panel in (C), each dataset was simulated from the best-fitting model to the real data, but with its gaze-modulation timecourse parameters forced to be the same for all neurons in all areas. The true timecourses had μ parameters making them occur before, simultaneous, or after gaze shifts (top, middle, bottom) and σ parameters making them be instantaneous, gradual, or very gradual (left, center, right). The model recovered close approximations of the true timecourse of gaze modulation (even when the true timecourse was very temporally sharp (left)) and correctly fitted similar timecourses to all three areas (all colored curves similar to the black curve).

(E) Model performance: fitted effect of gaze modulation for real and simulated data. Fits are shown for the real data (*left*), a simulated dataset generated from the best-fitting model to the real data (*middle*), and a second simulated dataset generated from the same model except that it has no gaze-modulation because w_{gain} and w_{offset} parameters are both set to zero for all neurons in all areas (*right*). Same plotting conventions as the histogram of fitted gains in the main text. Fitting results are displayed for all neurons that were significantly modulated by gaze in the real data.

The fit to the real data indicates that gaze-modulation often includes changes in response gain, typically consisting of an increase in gain but with some variability across neurons (left). The fits to the simulated data indicate that the model-fitting procedure is effective at recovering the true effect of gaze-modulation. Thus, in a simulation where the true gain changes are known to be equal to the distribution on the left, the fits accurately recover a close approximation of that distribution of gain changes (compare *middle* vs *left*), including a similarly significantly positive mean gain change (*bottom*). Likewise, in a simulation where there is known to be no true gaze-modulation, the fits accurately indicate that all neurons have little or no change in gain (*right*) and hence that the mean gain change is not significantly different from zero (*bottom*).

(F) Model performance: fitted effect of gaze modulation for simulated data with a variety of true effects. *Left*: distribution of gain changes fit to single neurons in simulations where the true $\log_2$(gain) was set to -1, 0, or 1 for all neurons (black dashed lines), meaning that neural responses are halved, unchanged, or doubled during gaze. The distributions of fitted gain changes are clustered around the true gain change (compare green distribution to black dashed lines). *Right*: relationship between true and mean fitted gain change across multiple simulations. The fitted gain changes (green) approximate the true gain changes (black dashed identity line).

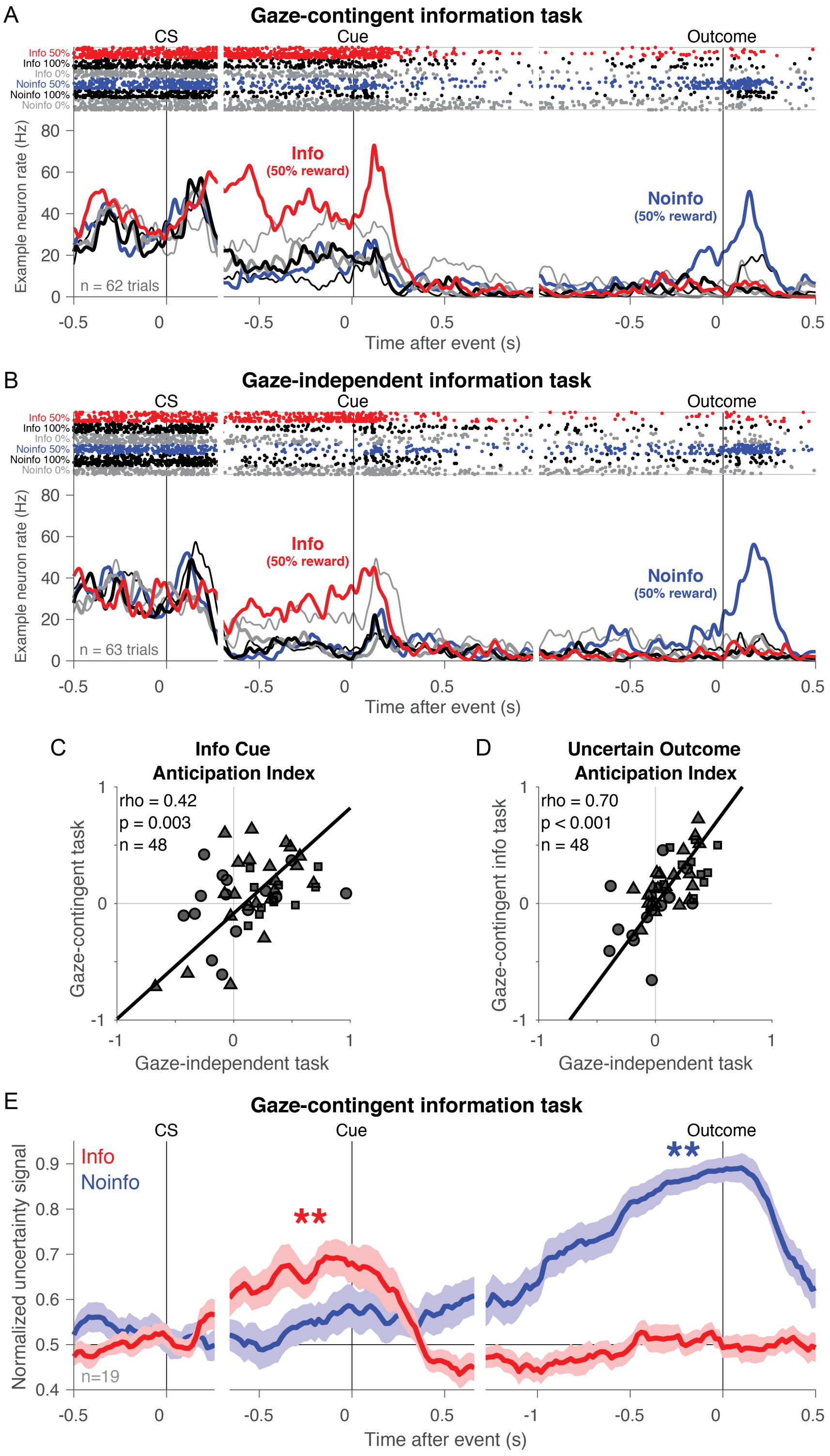

Supplementary Figure 9. Information-anticipatory signals are present when access to information is contingent on gaze.

(A,B) Activity of an example ACC neuron recorded in both the gaze-contingent information task (A) and the gaze-independent information task (B). Colored lines of different thicknesses indicate activity on Info trials (red thick, black thick, gray thick: 50%, 100%, and 0% reward) and Noinfo trials (blue thick, black thin, gray thin: 50%, 100%, and 0% reward). This neuron had

similar information-related activity in both tasks, with clear information-anticipatory activity during the CS period in both tasks. Notably, in anticipation of cue onset, its firing rate on Info 50% trials was higher than its firing rates on Noinfo 50% trials, on Info Certain trials, and on Noinfo Certain trials ($p = 0.0047$, $p = 0.0044$, $p = 0.0138$ respectively in the gaze-contingent task, and $p = 0.0011$, $p = 0.0002$, and $p = 0.0042$ in the gaze-independent task).
(C,D) Coding indexes are highly similar in the two tasks. Shown are the correlations between the gaze-contingent task and gaze-independent task measurements of the Info Cue Anticipation Index (C) and Uncertain Outcome Anticipation Index (D). Both are significantly positively correlated. Symbols indicate areas (triangle, square, and circle indicate ACC, icbDS, and Pal). For completeness and to any potential for avoid selection bias, this analysis includes all neurons recorded in both tasks; we obtained similar results if analyzing only neurons with significant uncertainty coding (n=40, Info Cue Anticipation Index rho = +0.35, $p = 0.026$; Uncertain Outcome Anticipation Index rho = +0.68, $p < 0.001$).
(E) Timecourse of normalized uncertainty signals in the gaze-contingent task for uncertainty coding neurons with significant Uncertain Outcome Anticipation Indexes. Same format as Fig 3C. Information-anticipatory activity is significantly present before the cue ($p = 0.01$, signed-rank test). Note that this data further confirms our finding (Fig 5A, Supplementary Fig 7) that neural information-anticipatory signals are not merely an artifact of allowing the animal to gaze freely at the stimulus. Information-anticipatory activity occurred during the CS period of the gaze-contingent task – a time when the animal was not allowed to gaze at the CS, because they were required to maintain fixation at the center of the screen until they received the go signal. Thus, information-anticipatory activity occurred when animals were anticipating information, even at times when they were not allowed to express their state of anticipation with an action.

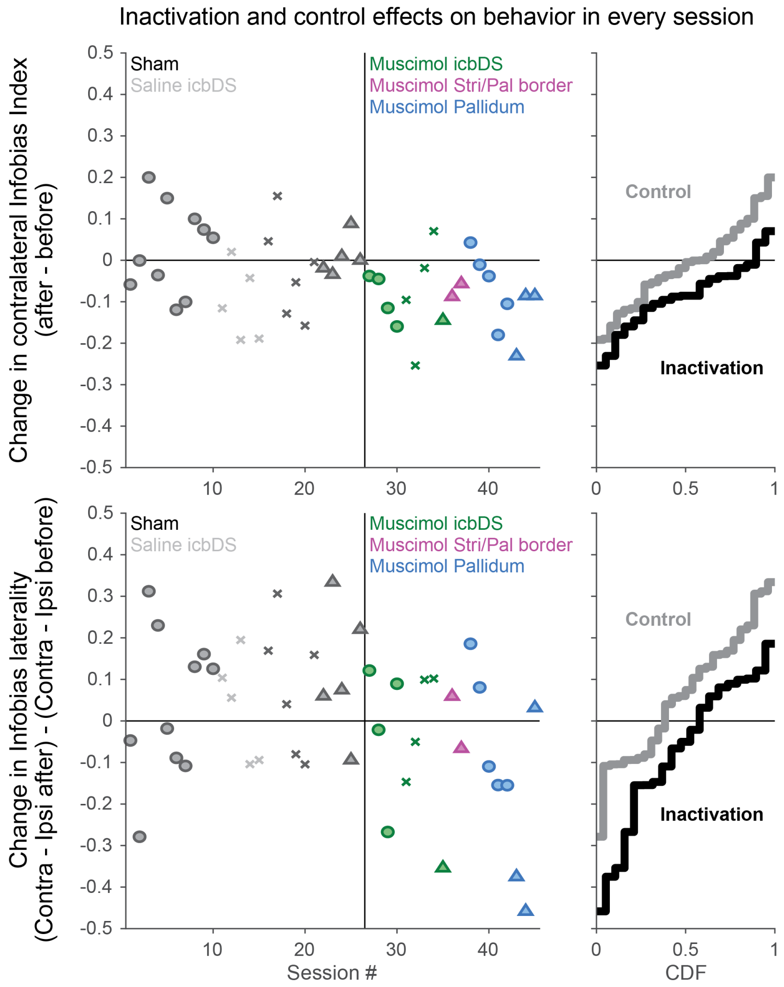

Supplementary Figure 10. Inactivation results from each session. Sessions are sorted by type (sham, saline, muscimol), animal (B, R, Z), and area (icbDS, Pal, Stri/Pal border region). The two Stri/Pal border region injections were excluded from our main analysis because they were done in a border region between the striatum and pallidum that was not clearly identifiable as strictly icbDS or Pal (between the ventral portion of icbDS and the dorsal portion of the anterior globus pallidus above the anterior commissure; Supplementary Table 2). However, they did not alter our results or conclusions if they were included in our analysis and are shown here for completeness. *Top:* change in Infobias Index for contralateral CSs. Left: data for single sessions. Right: cumulative distributions of the data plotted on the left. Inactivations resulted in a more negative change in Infobias Index relative to control (as shown in Fig 7). *Bottom:* change in laterality of Infobias Index (difference between Infobias Index computed for contralateral vs ipsilateral CSs). Inactivations resulted in the laterality of information seeking behavior being shifted more negatively (i.e. away from the contralateral side) relative to control (as shown in Supplementary Fig 11C).

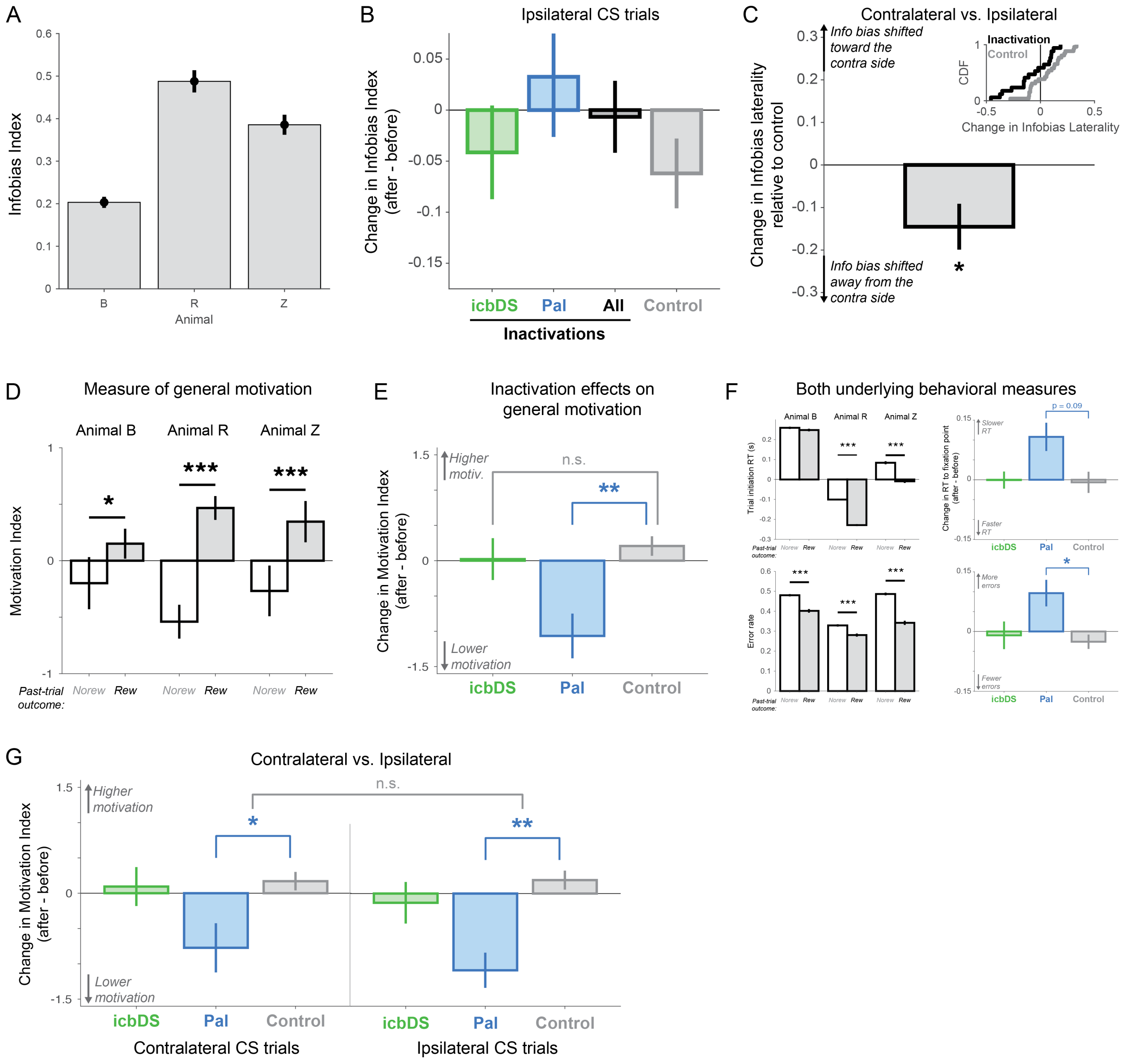

Supplementary Figure 11. Inactivation effects on laterality of information seeking behavior, general motivation, and effects in each session

(A) Mean Infobias Index in each animal. All animals had significantly positive Infobias Indexes in the mean over all sessions shown here and in all individual sessions (n=43/43; all $p < 0.05$, permutation tests). Error bars are ± 1 SE.

(B) No significant change in Infobias Indexes for ipsilateral CSs. Same format as Fig 7C, but for ipsilateral CSs. There is no significant change resulting from inactivations and no significant difference between inactivations and control.

(C) Inactivations change the laterality of information seeking behavior relative to control sessions. *Inset*: cumulative distributions of change in infobias laterality for inactivations (black) and controls (gray). Change in infobias laterality was quantified as: (change in Infobias Index for contralateral CSs) – (change in Infobias Index for ipsilateral CSs). *Bar plot*: difference in the change in infobias laterality between inactivations and controls. Error bars are ± bootstrap SE.

The change in infobias laterality was more negative for inactivations than controls ($p = 0.019$, permutation test), indicating that information seeking behavior was shifted away from the contralateral side relative to controls.
(D-G) Inactivation effects on general motivation and reward responsiveness. We quantified the animal's motivation to perform the task using two measures of general motivation that have been used in previous studies: the response time to initiate the trial on correctly performed trials, and the probability of making an error during the trial [2-4] (F). To summarize these measures and gain statistical power to detect any potential small effect of inactivation on motivation, we created a composite Motivation Index (D,E) pooling these measures by z-scoring each measure within each animal, averaging the two measures within each session, and then flipping its sign (so that positive Motivation Index indicates higher motivation to perform the task).
(D) Mean Motivation Index for each animal, plotted separately for trials in which the previous trial was rewarded (black) or non-rewarded (white). If the Motivation Index is a valid indicator of the animal's motivation then we expect it to indicate that most animals are more motivated to perform the task after receiving rewards. This result is commonly reported in monkeys unless tasks are designed to specifically encourage the opposite behavior [5]. Indeed, this was the case. By definition the mean Motivation Index over all trials is near zero, but all animals had a significantly higher Motivation Index after receiving reward than no reward (rank-sum tests, all $p < 0.05$).
(E) Mean change in Motivation Index (after – before), plotted separately for icbDS inactivation, Pal inactivation, and control sessions (colored bars). During control sessions there was no significant change in measures of motivation. icbDS inactivation sessions also had no apparent change in motivation in any measure, and their change in Motivation Index was not significantly different from control ($p = 0.414$, permutation test). Pal inactivation sessions had a trend for reduced motivation and their change in Motivation Index was significantly different from control ($p = 0.008$, permutation test).
(F) Similar results occurred for both individual motivational measures. When the past trial was rewarded, animals had faster trial initiation RTs and lower error rates (rank-sum tests, all $p < 0.001$ except for the trial initiation RT in animal B). Conversely, Pal inactivations tended to induce slower trial initiation RTs and higher error rates relative to control sessions, as either significant effects or non-significant trends (fixation RT, $p = 0.092$; error rate, $p = 0.018$). Note that the error rate includes both trials while the animal failed to fixate and trials when the animal fixated but then broke fixation. We obtained similar results when considering either one of these two types of errors individually (only failures to fixate, $p = 0.028$; only fixation breaks, $p = 0.043$). Note that the trial initiation RTs could be negative. This occurred when the animal gazed at the fixation window in an anticipatory manner before the fixation point appeared. This behavior appeared to reflect a situation in which the animal was highly motivated to complete the task, animals did this most frequently if they had received a reward on the previous trial (especially in animals R and Z), and this caused the trial to be initiated at the earliest possible time. This property of the data was not critical for our results; we obtained the same pattern of results in this figure if we enforced non-negative trial initiation RTs by setting all negative RTs to 0.
(G) Similar results occurred for both locations in space. On both contralateral CS trials and ipsilateral CS trials, the Motivation Index was unaffected by icbDS inactivation (contra $p = 0.67$, ipsi $p = 0.253$, no significant difference between them, $p = 0.573$) and was similarly and

significantly reduced by Pal inactivation (contra $p = 0.028$, ipsi $p = 0.004$, no significant difference between them, $p = 0.170$; permutation tests).

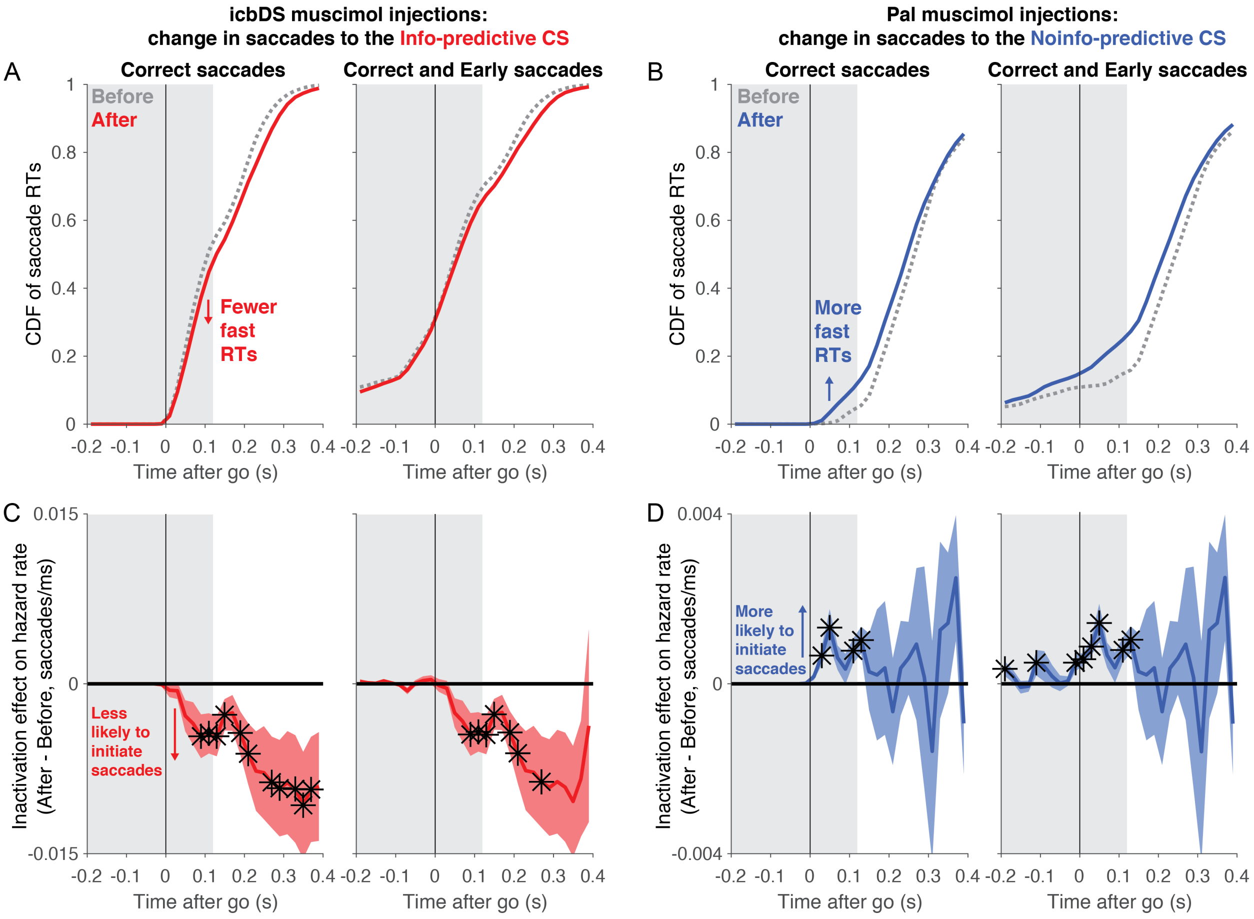

Supplementary Figure 12. Inactivation of icbDS and Pal influences anticipatory behavior. Our analysis thus far showed that inactivations of icbDS and Pal altered saccadic response times to Info and Noinfo CSs, respectively. Here we investigate in more detail how these inactivations affected saccade generation and when in the trial they had their effects. In particular, did they alter anticipatory saccades the animals generated while awaiting the go signal?

To address this, we first observed that the histograms of saccadic RTs permitted a relatively clear distinction between times in the trial when saccades were predominantly anticipatory vs. reactive. In the example histogram in Fig 7B, the initial ~120 ms after the go signal had a relatively low rate of responses, which was stable or gradually increasing over time. This is suggestive of anticipatory saccades, prepared based on the animal's internal estimate of when the go signal would occur. Then at ~120-150 ms there was a sudden, sharp increase in the response rate. This is suggestive of reactive saccades, generated as a reaction to the onset of the go signal. To quantify this distinction, we wanted to estimate the time after the go signal when behavior switched from anticipatory to reactive. Therefore, we made a conservative estimate of the minimal reactive RT. We built a histogram of all contralateral RTs and estimated the onset of the sharp peak by finding the millisecond after the go signal at which there was the largest difference between the frequency of saccades in the next 15 ms compared to the previous 15 ms. To be conservative, we then subtracted 15 ms from this estimate to ensure that it excluded the great majority of reactive saccades. The resulting estimates of the minimal reactive RT in this task

were reliable and consistent across animals (124 ± 2 ms, 119 ± 2 ms, 124 ± 1 ms in animals B, R, and Z, respectively; mean estimate ± bootstrap standard error).

Therefore, in this analysis we examined the timecourse of inactivation effects on saccade generation and whether these effects were present at times when saccades were predominantly anticipatory (< 120 ms after the go signal, gray shaded area in panels A-D). We examined inactivation effects on the cumulative distribution of RTs across time (**panels A,B**) and for a more precise measurement, we quantified their effects on the instantaneous probability of saccade initiation (also known as the hazard rate), which is more direct measure of perturbation effects on behavior at each moment in time[6] (**panels C,D**).

We performed this analysis on all correct trials (**left part of panels A-D**). To gain further insight into anticipatory behavior, we repeated this analysis on all correct trials plus error trials in which the animal made a premature saccade to the CS (**right part of panels A-D**, error saccades coded as 'negative RTs' because the saccade occurred before the go signal). Note that our hypothesis – that icbDS and Pal activity affect the motivation to seek information – does not make a strong prediction about whether inactivations should affect the likelihood or timing of premature saccade errors. For instance, if a manipulation increases the motivation to seek information, this could *increase* the rate of premature saccades (by making the Info CS more attractive), or *decrease* the rate of premature saccades (by making the animal more motivated to complete each trial correctly in order to obtain information), or cause *no change* in premature saccades (e.g. by both effects counterbalancing each other). Nonetheless, examining these trials may give further insight into inactivation effects on anticipatory behavior.

(A) Cumulative distribution of saccadic response times to the contralateral Info CSs before (gray) and after (red) icbDS inactivation, for all correct trials (left) and correct trials plus premature saccades (right). As expected from our findings in Fig 7, the inactivation shifted the RT distribution to have fewer fast RTs (red < gray). The CDFs visibly diverged within the first 120 ms after the go signal (gray shaded area), suggestive of an effect on anticipatory behavior.
(B) Same, for saccades to the contralateral Noinfo CS before (gray) and after (blue) Pal inactivation. As expected, the inactivation shifted the RT distribution to have more fast RTs (blue > gray). The CDFs visibly diverged within the first 120 ms after the go signal, suggestive of an effect on anticipatory behavior.
(C,D) Inactivation effects on the instantaneous probability of saccade initiation (i.e. the hazard rate) as a function of time relative to the go signal. This was calculated as follows. In each session and each before/after condition, we calculated the probability distribution of contralateral RTs with 1 ms precision and then smoothed it with a causal exponential kernel ($\tau = 30$ ms; causal smoothing ensures that estimates of effects on presumed anticipatory behavior (RTs < 120 ms) are not influenced by later events (RTs > 120 ms)). We then calculated the hazard rate in the standard manner as the probability of a response at each point in time given that a response has not yet occurred (i.e. $\text{hazardrate}(t) = p(RT = t) / p(RT \geq t)$). Finally, we calculated the inactivation effect on the hazard rate in each session as (hazard rate after – hazard rate before) and binned the results in 20 ms time bins. Plotted is the mean ± SE of the single session effects. Black asterisks indicate time points with significant effects (signed-rank test, $p < 0.05$).
(C) icbDS inactivation effect on instantaneous probability of saccades to the Info CS (n=9 sessions). After inactivation there was a substantial reduction in saccade probability. In the

analysis of correct trials (left) this effect started almost immediately after the go signal and continued for several hundred milliseconds. Notably, the effect first reached significance in the time bins starting at 80, 100, 120, and 140 ms after the go signal, overlapping with the gray shaded area. No significant effects were found at these or nearby times in the analogous analyses of control sessions, or for ipsilateral saccades on inactivation sessions. Similar results occurred in the analysis that included premature saccades (right). This suggests an effect on anticipatory saccades.

(D) Pal inactivation effect on instantaneous probability of saccades to the Noinfo CS (n=8 sessions). After inactivation there was a substantial increase in saccade probability. In the analysis of correct trials (left) this effect reached significance almost immediately after the go signal and at later times as well (time bins starting at 20, 40, 100, and 120 ms), suggestive of anticipatory behavior. No significant effects were found at these times or nearby times in the analogous analyses of control sessions, or for ipsilateral saccades on inactivation sessions. In the analysis including premature saccades (right), this effect also reached significance at some times before and adjacent to the go signal (time bins starting at -200, -120, -20, 0, and 20 ms). This suggests an effect on anticipatory saccades.

			#tasks recorded			*standard uncertainty tasks*					*information tasks*			
**Animal**	**Area**	**#cells**	**1**	**2**	**3**	**A**	**B**	**C**	**D**	**E**	**IA**	**IB**	**IC**	**ID**
All	All	484	423	53	8	140	112	87	25	25	77	67	42	36
	ACC	222	204	12	6	52	33	78	15		22	24	42	
	icbDS	127	103	23	1	63	49	9		7	24	24		
	Pal	135	116	18	1	25	30		10	18	31	19		36
B	All	204	174	25	5	56	45	45	25		22	22	42	
	ACC	113	101	8	4	12	9	36	15		13	13	42	
	icbDS	58	44	13	1	34	29	9			1	1		
	Pal	33	29	4		10	7		10		8	8		
R	All	117	104	13		27	12			25	27	15		36
	ACC	24	23	1		17	8							
	icbDS	22	21	1		6				7	10	10		
	Pal	71	60	11		4	4			18	17	5		36
Z	All	114	97	14	3	44	18	42			28	30		
	ACC	85	80	3	2	23	16	42			9	11		
	icbDS	22	14	8		16	1				13	13		
	Pal	7	3	3	1	5	1				6	6		
W	All	49	48	1		13	37							
	icbDS	25	24	1		7	19							
	Pal	24	24			6	18							

Supplementary Table 1. Neural dataset from each animal and task. Shown for each animal (B, R, Z, and W) and area (ACC, icbDS, and Pal) are the total number of neurons recorded, the number of neurons recorded in 1, 2, or 3 different tasks, and the number of neurons recorded in each of the tasks (standard uncertainty tasks A-E and information tasks IA-ID).

				Injection site relative to AC (mm)		
**Animal**	**Treatment**	**Volume (µL)**	**Area**	**Anterior**	**Lateral**	**Superior**
B	Muscimol	2	icbDS	3.5	6.9	2.7
B	Muscimol	2.5	icbDS	7.5	6.9	2.7
B	Muscimol	2	icbDS	6.5	6.9	2.8
B	Muscimol	1	icbDS	6.5	6.6	2.4
R	Muscimol	3	icbDS	5	9.5	2.2
R	Muscimol	2	icbDS	3	10.2	1.4
R	Muscimol	2	icbDS	3	10.2	1.4
R	Muscimol	2	icbDS	4	9.7	0.8
Z	Muscimol	1.5	icbDS	4	10.4	0.5
Z	Muscimol	2.5	Border	1	6.2	3.3
Z	Muscimol	2	Border	2	6.8	2.4
B	Muscimol	1.4	Pal	-0.5	9.1	-5.4
B	Muscimol	1	Pal	-0.5	8.2	-5
B	Muscimol	0.8	Pal	-1.5	9.8	-6.2
B	Muscimol	0.8	Pal	-1.5	9.8	-6.2
B	Muscimol	0.545	Pal	0.5	8.4	-3.2
Z	Muscimol	0.545	Pal	-1	7.9	-6.2
Z	Muscimol	0.636	Pal	-1	7.9	-6.2
Z	Muscimol	0.636	Pal	-1	7.9	-6.2
R	Saline	2	icbDS	4	9.7	0.8
R	Saline	2	icbDS	3	9.9	1
R	Saline	2	icbDS	3	9.1	1.6
R	Saline	2	icbDS	3	11.1	1.1
R	Saline	2	icbDS	4	11	0.9

Supplementary Table 2. Inactivation parameters from each session. Shown are each session's animal, treatment (muscimol or saline), volume injected (uL), area injected (icbDS, Pal, or two sites that were at an intermediate location on the Border between striatum and pallidum; Supplementary Fig 10), and reconstructed coordinates of the injection site (same coordinate system as in Supplementary Fig 2, i.e. positions are in mm and are relative to the midline superior tip of the anterior commissure).

Supplementary Note 1: further tests of graded uncertainty coding

Consistent with graded coding of uncertainty, in standard uncertainty tasks the network's uncertainty-related activity before outcome delivery was strongest for maximal uncertainty (50% CS), lower for intermediate uncertainty (25% and 75% CSs), and lowest for minimal uncertainty (0% and 100% CSs). Further consistent with conventional measures of uncertainty, this relationship had a highly nonlinear inverted-U shape (Fig 1E), such that neural signals increased much more when going from minimal to intermediate uncertainty than when going from intermediate to maximal uncertainty. For instance, the standard deviation of the reward distribution for minimal, intermediate, and maximal uncertainty is 0, 0.108, and 0.125 mL juice. Thus, if neuronal activity was linearly related to standard deviation, then we would predict that the difference in neural activity between minimal and intermediate uncertainty should be 6.5 times larger than the difference in neural activity between intermediate and maximal uncertainty (0.108 vs 0.017). Indeed, the average activity of uncertainty coding neurons in standard uncertainty tasks followed a similar pattern to this theoretical prediction: the difference in normalized activity between minimal and intermediate uncertainty was 5.6 times larger than the difference between intermediate and maximal uncertainty ($n=165$, 0.853 ± 0.08 vs. 0.153 ± 0.04).

To further test whether the network encoded uncertainty in a graded manner during standard uncertainty tasks, we asked whether the network responded significantly more strongly to the maximally uncertain CS (50% reward) than to each other individual CS. Indeed, the average normalized activity before outcome delivery was significantly stronger for the 50% CS than any other CS. This was true regardless of whether this test was done using all uncertainty coding neurons ($n=165$, 50% CS vs 0, 25, 75, 100% CSs: $p < 0.0001$, $p = 0.026$, $p = 0.005$, $p < 0.0001$, signed-rank tests) or using the subset of neurons with significantly different average responses to the 50% CS than to pooled data from 25 and 75% CSs ($n=32$, $p < 0.0001$, $p < 0.0001$, $p = 0.0002$, $p < 0.0001$).

Finally, we formally tested whether the network's activity was better fit as an inverted-U function of reward probability or by a simple binary distinction between certain vs. uncertain rewards. Specifically, we fit each neuron's normalized activity on each trial before the outcome using linear regression. To represent the hypothesis of an inverted-U, we used a model with three regressors: a constant, the probability of reward (i.e. a linear effect of reward probability), and the standard deviation of reward (i.e. an inverted-U effect of reward probability). To represent the hypothesis of binary uncertainty coding, we used a model that was the same except that the third regressor was a simple binary variable that was 0 for certain rewards and 1 for uncertain rewards. Since both models have the same number of parameters their fitting quality can be compared using their likelihoods. Therefore, for each model we calculated the log likelihood of the entire dataset by summing the log likelihoods of its fits to the individual neurons. We then compared the two models by calculating the difference between their log likelihoods. Finally, we tested whether the model comparison was significant by calculating the bootstrap 99% confidence interval of the difference between the log likelihoods, and tested whether the confidence interval included zero (i.e. indicating that the two models may fit similarly well) or excluded zero (i.e. indicating that one model fits better than the other model). The results showed that the inverted-U model fit the data considerably better than the binary uncertainty model

(n=165, log likelihood difference = 36.6 favoring the inverted-U model, bootstrap 99% CI = [12.3,63.6]).

Supplementary Note 2: design and logic of perturbation experiments

Here we detail two methodological points: the reasoning for perturbing the targeted brain structures and the logic of the experimental design of perturbations and controls.

Targeting brain structures for perturbation

We targeted icbDS and Pal for perturbation because, by combining known anatomy and physiology with our own anatomical and neurophysiological findings, we were able to make strong hypotheses about how their activity may be causally linked to information seeking behavior, and hence were able to have a clear framework for interpreting the results. While our results also indicate a link between ACC and information seeking, our findings do not allow us to make a firm hypothesis about the effects of perturbing that area and what it would teach us about the underlying neural mechanisms. Specifically:

(1) Our data suggest that Pal and icbDS are well-positioned to influence the network's access to information-related signals and generation of behavioral output. By contrast, even if ACC was inactivated, our data suggest that there would likely be an intact pathway for information signals to reach icbDS and Pal and influence behavior. Notably, our latency analysis shows that (rough) uncertainty signals appear at the shortest latency in Pal, our anatomical data indicates that this could be transmitted directly to icbDS by Pal→icbDS projections, and our gaze-modulation analysis indicates that icbDS and Pal change their activity significantly closer to the time of upcoming gaze shifts than ACC, suggesting a more proximal link to behavior. Taken together, our data suggests that ACC activity may be well-suited for a supervisory role in information anticipatory behavior, but that even without ACC, icbDS and Pal may still be capable of generating simple, rough information-anticipatory signals and using them to guide behavior – which could well be sufficient to perform the task used in perturbation experiments.

(2) Our study, and our model of perturbation effects on the circuit, is focused specifically on the subset of neurons that encode signals related to uncertainty. This allows us to make clear predictions for icbDS perturbations, since icbDS uncertainty-related neurons have very consistent response patterns (i.e. exclusively excitatory information signals with a relatively consistent timecourse across neurons). This also allows us to make fairly clear predictions for Pal perturbations. Pal has more diverse responses, and indeed, Pal perturbations also produced significant effects on measures of the animal's level of general motivation (e.g. error rates). However, these are known effects that have been produced in previous studies using Pal inactivations [4,7-12]. Hence, we designed our task so these would not confound our key test of information seeking. By contrast, ACC contains a diverse array of neurons related to different cognitive and motivational functions, such as the subjective value of an option, specific task-relevant features of options, rewards, punishments, etc., most of which can be encoded with either excitations or inhibitions (as shown by studies from many groups [13-18] including our own [19]). This makes it difficult to predict what side-effects to expect, especially as ACC inactivations have been rarely reported in the primate literature. Therefore, if ACC perturbations altered our measure of information seeking, it could also have complex side-effects on motivated behavior that make the results difficult to interpret.

(3) Dorsal striatum and Pal have projections within the same hemisphere. Thus, perturbations would be expected to produce effects that are (to a first order) localized to a single side of the brain, and hence preferentially affect contralateral behaviors. This is necessary for the logic of our experiments, as the comparison of 'contra' vs 'ipsi' conditions is a vital control to show that the perturbations have a specific effect on localized brain circuitry rather than a generalized effect on all behavior. By contrast, ACC projects to both hemispheres, including its own counterpart in the opposite hemisphere[20], and these fiber pathways are capable of transferring neural activation between hemispheres[21]. Perturbations would therefore potentially alter both hemispheres, perhaps in distinct manners, making it more difficult to form a clear hypothesis about the results.

Design of perturbation experiments and controls

Our study was designed so that the key logic of our conclusions is based primarily on measuring perturbation effects by comparison with control conditions collected from the same session and comparison between different types of perturbations. This is a common approach used in systems neuroscience, especially in experiments using animal models and behaviors that allow the use of a task with control conditions such as comparisons of behavior before vs. after perturbation, comparison of effects on different classes of sensory stimuli or motor responses, and/or comparison of perturbations in different brain areas (e.g. [4,12,22-27]). In our case, we employ all of these types of controls. Specifically, our study was designed for (A) testing the presence and validity of perturbation effects via internal controls, by comparing conditions within each experiment and testing whether they have specific relationships predicted by our hypothesis (before vs. after perturbation, contralateral vs. ipsilateral CS location, CS-directed vs. trial-initiating saccades), (B) testing the regional specificity of effects, via comparing perturbations in the two brain areas and testing whether they have the specific, distinct effects predicted by our hypothesis (icbDS vs. Pal). Below, we set out this logic in greater detail.

(1) We must test whether effects on behavior are caused by the perturbation – i.e. by the injection of a substance into the brain. As an alternative, behaviors may be merely generalized side-effects caused by the experimental apparatus for injection (e.g. the weight of the apparatus on the head causing attention to be biased to a specific location in space). We ruled this out by computing all key behavioral measures as differences between behavior both before vs. after the injection. Such effects cannot be generalized effects of the apparatus because it was present throughout the entire experiment. As another alternative, behaviors may change due to generalized effects of time (e.g. behavior changing over the course of a session as the animal becomes satiated). We ruled this out in two manners. First, we used a comparison between different types of perturbations. Perturbation experiments in icbDS and Pal were carried out using the same experimental timecourse, so any main effect of time should occur equally in the two datasets. Instead, perturbing icbDS vs. Pal resulted in specific, significantly different effects on behavior that were predicted by our hypothesis. Second, as an additional test, we carried out sham injection experiments that followed exactly the same experimental timecourse as muscimol injections, so any main effect of time should occur equally in these datasets as well. Instead, there was no significant 'before vs. after' difference in our measure of information seeking during sham sessions, and sham sessions were significantly different from muscimol sessions.

Thus, we conclude that the measured perturbation effects were indeed caused by the perturbation.

(2) We must test whether effects are caused by perturbing the targeted area. As an alternative, effects may be merely non-specific effects of perturbing the brain, or the local region of the basal ganglia, rather than of perturbing the specifically targeted nuclei. To rule this out, we used two controls. First, we compared perturbation effects between contralateral vs ipsilateral space. If perturbations caused a generalized effect on brain function then it would have similar effects for saccades to either side. Instead, perturbation effects were significantly stronger in space contralateral to the perturbation target, as predicted by our hypothesis. Second, we compared perturbation effects between the two targeted areas. If effects were simply due to perturbing the rough vicinity of this area of the basal ganglia *per se*, then we would expect similar effects from perturbing icbDS vs Pal. Instead, perturbing the two targets impaired information seeking in specific, opposite manners as predicted by our hypothesis.

(3) We must test whether effects are caused by perturbing information seeking behavior. As an alternative, effects may have been due to a more general effect on motivation (e.g. saccades becoming faster due to higher general motivation to seek juice). To rule this out, we used two internal controls. We computed two independent measures of general motivation – latency to saccade to the fixation point to initiate the trial and probability of completing the trial successfully. If perturbations affected information seeking due to a generalized effect on motivation, they should have a consistent effect on these measures. Instead, we found that this was not the case: icbDS perturbations slowed saccades to the Info CS despite no reduction in general motivation, while Pal perturbations speeded saccades to the Noinfo CS despite no enhancement of general motivation (in fact, this speeding of saccades occurred despite a modest reduction of general motivation, consistent with the results of previous Pal studies).

Thus, we conclude that the perturbation effects were caused by the perturbations having an effect on the targeted areas and thereby altering information seeking behavior. Future research may further interrogate these areas to discover the specific biochemical process by which our perturbations affected icbDS and Pal neurons and information seeking behavior. Our results are consistent with injections having their effect by the classic action of muscimol via agonizing $GABA_A$ receptors and hence inhibiting striatal and pallidal neurons. This is based the fact that muscimol is a highly potent and selective $GABA_A$ agonist[28,29] and $GABA_A$ receptors potently inhibit striatal and pallidal neurons [30]. In addition, there are two lines of evidence from our study supporting this possibility. First, the observed effects on information seeking behavior are as predicted from a perturbation that has its effect via inactivating nearby neurons, including: reduction in measures of information seeking from both icbDS and Pal injections, the specific manner in which icbDS injections affected saccades to the Info CS, and the specific manner in which Pal injections affected saccades to the Noinfo CS (as illustrated by our model and its predictions in Fig 7). Second, the observed effect of muscimol injection in Pal causing a modest reduction in measures of general motivation (e.g. error rates), which is consistent with previous studies which injected muscimol into Pal and compared it to other substances [7,8,11].

REFERENCES

1 White, J. K. & Monosov, I. E. Neurons in the primate dorsal striatum signal the uncertainty of object-reward associations. *Nat Commun* **7** (2016).

2 Minamimoto, T., La Camera, G. & Richmond, B. J. Measuring and modeling the interaction among reward size, delay to reward, and satiation level on motivation in monkeys. *J Neurophysiol* **101**, 437-447 (2009).

3 Bromberg-Martin, E. S., Matsumoto, M., Nakahara, H. & Hikosaka, O. Multiple timescales of memory in lateral habenula and dopamine neurons. *Neuron* **67**, 499-510 (2010).

4 Tachibana, Y. & Hikosaka, O. The Primate Ventral Pallidum Encodes Expected Reward Value and Regulates Motor Action. *Neuron* **76**, 826-837 (2012).

5 Nakahara, H., Itoh, H., Kawagoe, R., Takikawa, Y. & Hikosaka, O. Dopamine neurons can represent context-dependent prediction error. *Neuron* **41**, 269-280 (2004).

6 Zimnik, A. J., Lara, A. H. & Churchland, M. M. Perturbation of Macaque Supplementary Motor Area Produces Context-Independent Changes in the Probability of Movement Initiation. *J Neurosci* **39**, 3217-3233 (2019).

7 Taha, S. A., Katsuura, Y., Noorvash, D., Seroussi, A. & Fields, H. L. Convergent, not serial, striatal and pallidal circuits regulate opioid-induced food intake. *Neuroscience* **161**, 718-733 (2009).

8 Shimura, T., Imaoka, H. & Yamamoto, T. Neurochemical modulation of ingestive behavior in the ventral pallidum. *Eur J Neurosci* **23**, 1596-1604 (2006).

9 Smith, K. S., Tindell, A. J., Aldridge, J. W. & Berridge, K. C. Ventral pallidum roles in reward and motivation. *Behav Brain Res* **196**, 155-167 (2009).

10 Root, D. H., Melendez, R. I., Zaborszky, L. & Napier, T. C. The ventral pallidum: Subregion-specific functional anatomy and roles in motivated behaviors. *Prog Neurobiol* **130**, 29-70 (2015).

11 Richard, J. M., Ambroggi, F., Janak, P. H. & Fields, H. L. Ventral Pallidum Neurons Encode Incentive Value and Promote Cue-Elicited Instrumental Actions. *Neuron* **90**, 1165-1173 (2016).

12 Fujimoto, A. *et al.* Signaling Incentive and Drive in the Primate Ventral Pallidum for Motivational Control of Goal-Directed Action. *J Neurosci* **39**, 1793-1804 (2019).

13 Rushworth, M. F. & Behrens, T. E. Choice, uncertainty and value in prefrontal and cingulate cortex. *Nat Neurosci* **11**, 389-397 (2008).

14 Heilbronner, S. R. & Hayden, B. Y. Dorsal Anterior Cingulate Cortex: A Bottom-Up View. *Annu Rev Neurosci* **39**, 149-170 (2016).

15 Otis, J. M. *et al.* Prefrontal cortex output circuits guide reward seeking through divergent cue encoding. *Nature* **543**, 103-107 (2017).

16 Hosokawa, T., Kennerley, S. W., Sloan, J. & Wallis, J. D. Single-neuron mechanisms underlying cost-benefit analysis in frontal cortex. *J Neurosci* **33**, 17385-17397 (2013).

17 Kennerley, S. W. & Wallis, J. D. Evaluating choices by single neurons in the frontal lobe: outcome value encoded across multiple decision variables. *Eur J Neurosci* **29**, 2061-2073 (2009).

18 Kennerley, S. W., Dahmubed, A. F., Lara, A. H. & Wallis, J. D. Neurons in the frontal lobe encode the value of multiple decision variables. *J Cogn Neurosci* **21**, 1162-1178 (2009).

19 Monosov, I. E. Anterior cingulate is a source of valence-specific information about value and uncertainty. *Nat Commun* **8** (2017).

20 Nimchinsky, E. A., Hof, P. R., Young, W. G. & Morrison, J. H. Neurochemical, morphologic, and laminar characterization of cortical projection neurons in the cingulate motor areas of the macaque monkey. *J Comp Neurol* **374**, 136-160 (1996).

21 Walker, J., Storch, G., Quach-Wong, B., Sonnenfeld, J. & Aaron, G. Propagation of epileptiform events across the corpus callosum in a cingulate cortical slice preparation. *PLoS One* **7**, e31415 (2012).

22 Shi, D., Friedman, H. R. & Bruce, C. J. Deficits in smooth-pursuit eye movements after muscimol inactivation within the primate's frontal eye field. *J Neurophysiol* **80**, 458-464 (1998).

23 Herman, J. P., Katz, L. N. & Krauzlis, R. J. Midbrain activity can explain perceptual decisions during an attention task. *Nat Neurosci* **21**, 1651-1655 (2018).

24 Bollimunta, A., Bogadhi, A. R. & Krauzlis, R. J. Comparing frontal eye field and superior colliculus contributions to covert spatial attention. *Nat Commun* **9**, 3553 (2018).

25 Rajalingham, R. & DiCarlo, J. J. Reversible Inactivation of Different Millimeter-Scale Regions of Primate IT Results in Different Patterns of Core Object Recognition Deficits. *Neuron* **102**, 493-505 e495 (2019).

26 Monosov, I. E., Sheinberg, D. L. & Thompson, K. G. The effects of prefrontal cortex inactivation on object responses of single neurons in the inferotemporal cortex during visual search. *J Neurosci* **31**, 15956-15961 (2011).

27 Kojima, Y. & Soetedjo, R. Elimination of the error signal in the superior colliculus impairs saccade motor learning. *Proc Natl Acad Sci U S A* **115**, E8987-E8995 (2018).

28 Andrews, P. R. & Johnston, G. A. GABA agonists and antagonists. *Biochem Pharmacol* **28**, 2697-2702 (1979).

29 Krall, J. *et al.* GABAA receptor partial agonists and antagonists: structure, binding mode, and pharmacology. *Adv Pharmacol* **72**, 201-227 (2015).

30 Goetz, T., Arslan, A., Wisden, W. & Wulff, P. GABA(A) receptors: structure and function in the basal ganglia. *Prog Brain Res* **160**, 21-41 (2007).
