# Supplementary material for: A neural network for information seeking
Source: Nat Commun. 2019 Nov 14;10:5168. doi: 10.1038/s41467-019-13135-z (PMC6856099; doi:10.1038/s41467-019-13135-z)
Supplement: Supplementary file 3 — Reporting Summary [file 41467_2019_13135_MOESM3_ESM.pdf]

nature research

Corresponding author(s): Ilya Monosov

Last updated by author(s): October 3, 2019

Reporting Summary

Nature Research wishes to improve the reproducibility of the work that we publish. This form provides structure for consistency and transparency in reporting. For further information on Nature Research policies, see Authors & Referees and the Editorial Policy Checklist.

Statistics

For all statistical analyses, confirm that the following items are present in the figure legend, table legend, main text, or Methods section.

n/a	Confirmed	
☐	☒	The exact sample size (n) for each experimental group/condition, given as a discrete number and unit of measurement
☐	☒	A statement on whether measurements were taken from distinct samples or whether the same sample was measured repeatedly
☐	☒	The statistical test(s) used AND whether they are one- or two-sided<br>*Only common tests should be described solely by name; describe more complex techniques in the Methods section.*
☐	☒	A description of all covariates tested
☐	☒	A description of any assumptions or corrections, such as tests of normality and adjustment for multiple comparisons
☐	☒	A full description of the statistical parameters including central tendency (e.g. means) or other basic estimates (e.g. regression coefficient) AND variation (e.g. standard deviation) or associated estimates of uncertainty (e.g. confidence intervals)
☐	☒	For null hypothesis testing, the test statistic (e.g. F, t, r) with confidence intervals, effect sizes, degrees of freedom and P value noted<br>*Give P values as exact values whenever suitable.*
☒	☐	For Bayesian analysis, information on the choice of priors and Markov chain Monte Carlo settings
☒	☐	For hierarchical and complex designs, identification of the appropriate level for tests and full reporting of outcomes
☐	☒	Estimates of effect sizes (e.g. Cohen's d, Pearson's r), indicating how they were calculated

Our web collection on statistics for biologists contains articles on many of the points above.

Software and code

Policy information about availability of computer code

Data collection	For behavioral task control we used Matlab R2015a (Mathworks) with Psychophysics Toolbox, Datapixx (VPixx Technologies), and EyeLink 1000 (SR Research). For electrophysiological recording we used AlphaLab SnR (AlphaOmega). For anatomical data collection we used Neurolucida (MBF Bioscience) and StereoInvestigator (Micro-BrightField).
Data analysis	We used Matlab R2017b (Mathworks) including the Statistics and Optimization toolboxes.

For manuscripts utilizing custom algorithms or software that are central to the research but not yet described in published literature, software must be made available to editors/reviewers. We strongly encourage code deposition in a community repository (e.g. GitHub). See the Nature Research guidelines for submitting code & software for further information.

Data

Policy information about availability of data

All manuscripts must include a data availability statement. This statement should provide the following information, where applicable:

- Accession codes, unique identifiers, or web links for publicly available datasets
- A list of figures that have associated raw data
- A description of any restrictions on data availability

The datasets generated during and/or analyzed during the current study are available from the corresponding author on reasonable request.

Field-specific reporting

Please select the one below that is the best fit for your research. If you are not sure, read the appropriate sections before making your selection.

☒ Life sciences ☐ Behavioural & social sciences ☐ Ecological, evolutionary & environmental sciences

For a reference copy of the document with all sections, see nature.com/documents/nr-reporting-summary-flat.pdf

Life sciences study design

All studies must disclose on these points even when the disclosure is negative.

Sample size	No statistical methods were used to predetermine sample sizes. We recorded a total of 484 neurons with uncertainty-related activity across three brain areas (ACC n=222, icbDS n=127, Pal n=135, typical numbers for single unit recording studies) in four monkeys (more than typical single unit electrophysiology studies which use 2-3 monkeys). Our goal was to collect sufficient data to characterize the presence of uncertainty signals in these areas and their relation to information anticipation and found a highly significant effect. We performed a total of 19 inactivation sessions across two brain aresa (icbDS n=9, Pal n=8, icbDS-Pal border n=2) and 26 control sessions in three animals. Our goal was to collect sufficient data to test whether inactivations affected information seeking behavior and found a highly significant effect.
Data exclusions	As described in Methods, n=6 Pal neurons were excluded from analysis of gaze behavior due to an error in configuring the eye tracker causing noisy measurements of gaze, and n=2 inactivation sessions were excluded from analysis due to being located on the border between icbDS and Pal.
Replication	As described in the main text and supplemental material, monkeys were trained and tested over a period of years producing consistent behavioral results, and key experimental results were found consistently in data collected using multiple tasks and/or multiple animals.
Randomization	No experimental groupings were used for this study.
Blinding	Not applicable because no grouping was performed.

Reporting for specific materials, systems and methods

We require information from authors about some types of materials, experimental systems and methods used in many studies. Here, indicate whether each material, system or method listed is relevant to your study. If you are not sure if a list item applies to your research, read the appropriate section before selecting a response.

Materials & experimental systems

n/a	Involved in the study	
☒	☐	Antibodies
☒	☐	Eukaryotic cell lines
☒	☐	Palaeontology
☐	☒	Animals and other organisms
☒	☐	Human research participants
☒	☐	Clinical data

Methods

n/a	Involved in the study	
☒	☐	ChIP-seq
☒	☐	Flow cytometry
☒	☐	MRI-based neuroimaging

Animals and other organisms

Policy information about studies involving animals; ARRIVE guidelines recommended for reporting animal research

Laboratory animals	Four male rhesus macaque monkeys (Macaca mulatta) ages 5-7.
Wild animals	Not applicable.
Field-collected samples	Not applicable.
Ethics oversight	All procedures conformed to the Guide for the Care and Use of Laboratory Animals. Electrophysiology and pharmacology were performed at Washington University and were approved by the Washington University Institutional Animal Care and Use Committee. Anatomy procedures were performed at the University of Rochester and were approved by the University Committee on Animal Resources at the University of Rochester.

Note that full information on the approval of the study protocol must also be provided in the manuscript.
